# *In vivo* HSC gene therapy enables sustained eCD4-Ig expression for SIV prevention

**DOI:** 10.1016/j.omta.2026.201683

**Published:** 2026-02-26

**Authors:** Chang Li, Anna K. Anderson, Anne-Sophie Kuhlmann, Veronica Nelson, Audrey Germond, Hongjie Wang, Aphrodite Georgakopoulou, Sucheol Gil, Jasmin Martinez-Reyes, Andrew Riker, Shruthi Shankar Raman, Jiho Kim, Philip Ng, Donna Palmer, Michael D. Alpert, Nickolas Skamangas, Charles Bailey, Tianling Ou, Christine M. Fennessey, Michael Farzan, Keith R. Jerome, Brandon F. Keele, Hans-Peter Kiem, André Lieber, John K. Bui

**Affiliations:** 1 Department of Medicine, Division of Medical Genetics, University of Washington, Seattle, WA 98195, USA; 2 Fred Hutchinson Cancer Center, Seattle, WA 98109, USA; 3 University of Washington, Washington National Primate Research Center, Seattle, WA 98195, USA; 4 PAI Life Sciences Inc., Seattle, WA 98102, USA; 5 Baylor College of Medicine, Molecular and Human Genetics, Houston, TX 77030, USA; 6 Emmune Inc, Cambridge, MA 02139, USA; 7 Boston Children’s Hospital, Boston, MA 02115, USA; 8 Broad Institute of MIT and Harvard, Cambridge, MA 02142, USA; 9 AIDS and Cancer Virus Program, Frederick National Laboratory for Cancer Research, Frederick, MD 21702, USA; 10 Department of Laboratory Medicine and Pathology, University of Washington, Seattle, WA 98195, USA; 11 Department of Medicine, Division of Allergy and Infectious Diseases, University of Washington, Seattle, WA 98195, USA

**Keywords:** hematopoietic stem cells, gene therapy, *in vivo*, mobilization, helper-dependent adenovirus vectors, rhesus macaques, HIV, SIV, eCD4-Ig

## Abstract

We aim to develop an *in vivo* hematopoietic stem cell (HSC) gene therapy approach for the prevention and control of HIV-1 infection. Toward this goal, we engineered helper-dependent adenovirus (HDAd) 6/3+ vectors to directly transduce HSCs *in vivo*, enabling progeny cells to secrete eCD4-Ig, a decoy protein that broadly neutralizes HIV/simian immunodeficiency virus (SIV) isolates by mimicking the primary viral receptor CD4 and coreceptors such as CCR5. In rhesus macaques, the HDAd 6/3+ platform achieved long-term expression of an enhanced eCD4-Ig variant (“eCD4-Ig-Emm06”) that retained potent neutralization efficacy *in vivo*. Transduced HSCs differentiated into lymphoid and myeloid lineages and trafficked to systemic tissues, with B cells emerging as a major source of eCD4Ig-Emm06. HDAd-eCD4Ig-Emm06-treated animals had significantly reduced splenic viral reservoirs, and the animal with the highest circulating levels of eCD4Ig-Emm06 exhibited fewer founder viruses, delayed onset to viremia, and lower plasma viral loads, demonstrating promise within this proof-of-concept study. Further improvements in protective efficacy may be achieved through approaches identified in this study, including lineage-specific expression, reduced immunogenicity, and efficient selection. These findings validate HDAd 6/3+ as a promising platform for durable gene-based delivery of biologic therapeutics and guide advancement of HSC gene therapy for HIV and other chronic infections.

## Graphical abstract

**Figure:**
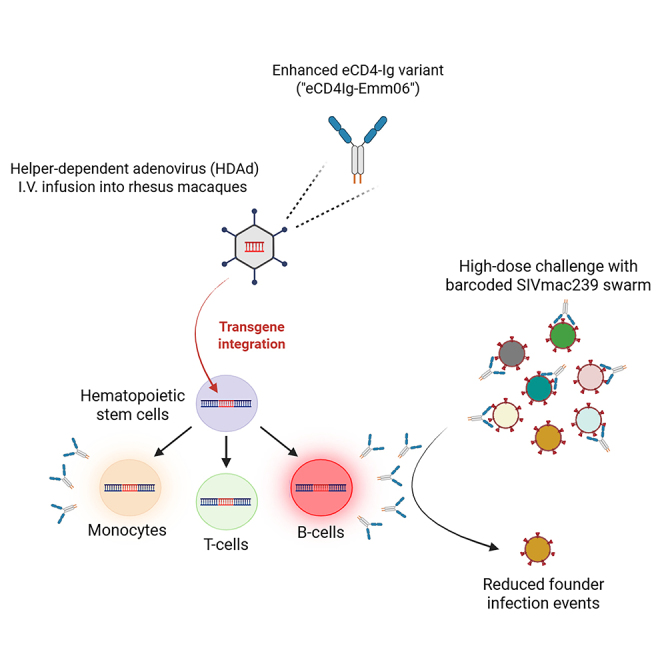

## Teaser

Li and colleagues develop a new HDAd 6/3+ vector for specific transduction of hematopoietic stem cells, enabling scalable and accessible therapeutic delivery. They validated this platform in mouse and nonhuman primate models, demonstrating sustained levels of eCD4-Ig for HIV/SIV prevention. B cells were the primary secretors, informing future gene therapy approaches.

## Introduction

Despite the availability of antiretroviral therapy (ART), HIV continues to pose a major global health challenge, with 40 million people living with HIV and continued transmission occurring globally.^1^ In the absence of an effective HIV vaccine, gene therapy-mediated delivery of eCD4-Ig has emerged as a promising strategy for HIV prevention and aligns with high-priority efforts toward a single-shot functional cure.^2^^,^^3^^,^^4^ eCD4-Ig is a chimeric protein that mimics HIV’s obligate entry receptors by combining the extracellular CD4 binding domain, IgG Fc region, and a coreceptor mimetic peptide.^2^^,^^3^^,^^5^ This structure enables eCD4-Ig to bind with high affinity to the HIV envelope, with exceptional breadth across HIV and simian immunodeficiency virus (SIV) isolates. In nonhuman primate (NHP) studies, eCD4-Ig conferred robust protection against high-dose SHIV and SIV challenges.^2^^,^^3^ The breadth, potency, and high genetic barrier to resistance of eCD4-Ig underscores its potential as a therapeutic candidate.

The helper-dependent adenovirus (HDAd) platform offers a gene therapy strategy for long-term delivery of eCD4-Ig that eliminates the need for repeated passive eCD4-Ig infusions, offering a scalable and accessible therapeutic strategy for middle- and low-income countries that face a disproportionate burden of HIV/AIDS.^1^^,^^6^^,^^7^^,^^8^^,^^9^^,^^10^^,^^11^^,^^12^^,^^13^ A promising HDAd approach enables stable expression by progeny cells using intravenous infusions to transduce hematopoietic stem cells (HSCs) *in vivo*.^7^^,^^9^^,^^14^^,^^15^^,^^16^^,^^17^ HDAd vector safety has been enhanced by removing viral coding sequences (“gutless” vectors) to render them replication incompetent and by co-administering cytokine prophylaxis to blunt innate immune responses. We have previously reported the use of HDAd 5/35++ vectors in murine and NHP models to achieve sustained expression of eCD4-Ig, with reduced immunogenicity compared to AAV-based approaches.^2^^,^^3^^,^^9^ While high levels of eCD4-Ig (∼20 μg/mL) confirmed the efficacy of our HDAd platform, both eCD4-Ig-treated and control animals showed similar susceptibility to SHIV.D infection, highlighting the need to further improve gene delivery and therapeutic potency.

In this study, we developed the optimized HDAd 6/3+ platform to deliver an enhanced variant of rhesus eCD4-Ig with improved stability and neutralization potency. To maximize therapeutic efficacy, we co-delivered TPST2 to support eCD4-Ig sulfation.^3^ Protective efficacy of *in vivo*-generated eCD4-Ig was assessed using sequential, escalating challenges with SIVmac239 to stringently measure the efficacy of our *in vivo* HDAd eCD4-Ig delivery approach.

## Results

### HDAd 6/3+ vectors for enhanced eCD4Ig-Emm06 delivery

**Figure 1:**
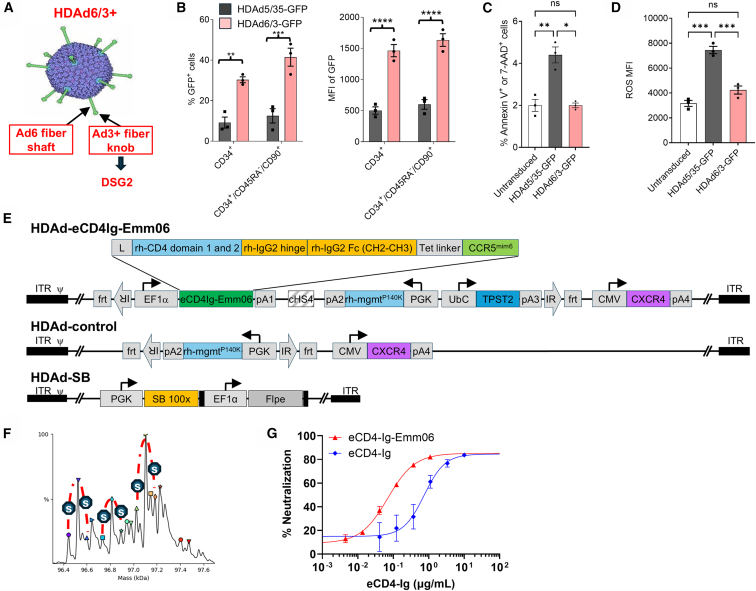
*In vitro* characterization of HDAd-eCD4Ig-Emm06 (A) Left: Schematic of HDAd structure. HDAd6/3+ vectors are derived from adenovirus serotype 6. These vectors incorporate the Ad6 fiber shaft and the DSG2 affinity-enhanced Ad3 fiber knob for liver detargeting and HSC targeting, respectively. (B–D) *In vitro* transduction of rhesus CD34+ cells (*n* = 3 donors) with HDAd6/35++ and HDAd6/3+ vectors expressing GFP. Infection was performed at an MOI of 4,000 viral particles (vp) per cell. (B) GFP expression measured in total CD34+ cells or in CD34+/CD45RA-/CD90+ cells at day 3 post-transduction. (C) Cell viability assessed by Annexin V and 7-AAD staining 24 h post-transduction using flow cytometry. (D) Reactive oxygen species (ROS) median fluorescence intensity (MFI) measured 24 h post-transduction. (E) HDAd vector structure. HDAd-eCD4Ig-Emm06: eCD4Ig-Emm06 contains the rhesus CD4 leader sequence, rhesus CD4 domains 1 and 2, the rhesus IgG2 hinge and Fc regions, and the CCR5 mimetic peptide (CCR5_mim_). The eCD4Ig-Emm06 construct is under the control of the ubiquitously active EF1α promoter. Additional functional cassettes are included for the expression of i) rhesus mgmt^P140K^ (to confer resistance against O^6^BG/BCNU), ii) rhesus tyrosylprotein sulfotransferase 2 (TPST2) (for sulfation of the CCR5_mim_ peptide), and iii) CXCR4 (for homing of mobilized HSCs to the bone marrow). frt: Flpe recombinase binding sites for circulation of the transposon, located between the two inverted repeats (IRs) recognized by SB100×. PGK, phoshoglycerate kinase promoter; cHS4, chicken globin insulator; UbC, ubiquitin C promoter; ITR, adenovirus inverted repeat; Ψ, adenovirus Ad6 packaging signal. HDAd-control lacks the eCD4Ig-Emm06 and TPST2 cassettes. Integration of transposons is facilitated by the HDAd-SB vector, which contains the SB100× and Flpe expression cassettes. (F) 239-FT cells were transduced with HDAd vectors expressing eCD4-Ig-Emm06 and TPST2. eCD4Ig-Emm06 was purified (see Figure S1A) and analyzed by intact protein mass spectrometry following PNGase F digestion for deglycosylation. Satellite peaks separated by ∼80 Da suggest sulfation, while major peaks likely reflect persistent O-linked glycosylation (see Figure S1B for intensity and D-score values). (G) SIVmac239 was incubated in duplicate with TZM-bl cells and varying concentrations of recombinant eCD4-Ig and eCD4-Ig-Emm06. Luciferase activity, indicating SIV infection, was measured 2 days post-infection. Percent neutralization was calculated as the difference in relative light units (RLU) between virus control (virus only) and test wells (virus and recombinant eCD4-Ig), divided by the difference in RLU between virus control and cell control (no virus) wells. Data are plotted as mean ± SEM. Statistical comparisons: Figure 1B, two-way ANOVA; Figures 1C and 1D, one-way ANOVA with Tukey’s correction for multiple comparisons. Significance is indicated as ∗*p* < 0.05, ∗∗*p* < 0.01, ∗∗∗*p* < 0.001, ∗∗∗∗*p* < 0.0001.

HDAd 6/3+ vectors were manufactured with the Ad6 serotype and affinity-enhanced Ad3 fiber knob domains and compared to our previously validated HDAd 5/35++ vectors, which possess the Ad5 serotype and Ad35 affinity-enhanced fiber knob domains (Figure 1A).^6^^,^^7^^,^^8^^,^^9^ The Ad6 capsid has lower seroprevalence in humans compared to Ad5, as reported by others^18^ and demonstrated in our prior work, with >90% of human serum samples containing anti-Ad5 antibodies, whereas only ∼15% contained anti-Ad6 antibodies.^6^ This reduced seroprevalence makes Ad6 a favorable candidate for *in vivo* gene therapy. Additionally, the Ad3 fiber knob enables more specific transduction of NHP HSCs through desmoglein 2 (DSG2), supporting its use in preclinical NHP models.^14^^,^^19^ The HDAd 6/3+ and 5/35++ vector pseudotypes were compared by generating vectors of each pseudotype expressing GFP and mgmt^P140K^ for selection and transducing rhesus HSCs at an MOI of 4,000 viral particles per cell, which approximates the *in vivo* transduction efficiency of GFP-expressing HDAd vectors based on previously published data.^14^^,^^20^ *In vitro*, the HDAd 6/3+ vectors achieved superior transduction efficiency in rhesus HSCs, including CD34^+^ and CD34^+^CD90^+^CD45RA^−^ subsets (*p* = 0.0017, *p* = 0.0002), the latter enriched for cells responsible for hematopoietic reconstitution (Figure 1B).^21^^,^^22^^,^^23^ Transgene expression in HSCs was likewise enhanced with HDAd 6/3+ vectors (*p* < 0.0001) (Figure 1B). HDAd 6/3+ was associated with reduced toxicity, as described by the percentage of dead cells (*p* = 0.0005) and production of reactive oxygen species (ROS) (*p* = 0.0217) (Figures 1C and 1D). With validated improvements in transduction efficiency and reduced toxicity of the HDAd 6/3+ platform, we generated HDAd vectors for *in vivo* delivery of eCD4-Ig in mouse and NHP models.

The HDAd-eCD4Ig-Emm06 vector was engineered to encode an integrating transgene cassette, with eCD4-Ig expression driven by the EF1α promoter and *mgmt*^*P140K*^ by the PGK promoter to enable selection of transduced cells. The ubiquitin C promoter was used to express *TPST2* to support eCD4-Ig sulfation (Figure 1E). We used a rhesus eCD4-Ig variant (‘eCD4Ig-Emm06’) containing the mim6 coreceptor mimetic sulfopeptide, substitutions in rhesus macaque CD4 that enhance stability, neutralization potency, and *in vivo* half-life (A55V, M69L, V128L, and V168L), and the half-life-extending “LS” substitutions in the rhesus IgG2 Fc.^24^^,^^25^^,^^26^ To enhance homing of transduced HSCs to the bone marrow, CXCR4 was transiently expressed using a non-integrating cassette driven by a CMV promoter, which is naturally silenced over time.^14^ The HDAd control vectors were designed similarly to the therapeutic vectors but omitted the eCD4Ig-Emm06 and TPST2 transgenes. The HDAd-SB vector encoded the *Sleeping Beauty* transposase system (SB100) to mediate integration of transgene cassettes.

To validate the HDAd vector’s capacity to generate functional eCD4Ig-Emm06, HDAd vectors were used to transduce 293T cells, and secreted eCD4-Ig was purified from the culture supernatant (Figure S1A). As 293T cells lack endogenous TPST2, any observed sulfation of eCD4-Ig can be attributed to the introduced TPST2 transgene.^27^ Intact protein mass spectrometry confirmed successful sulfation by TPST2 co-expression, indicated by satellite peaks separated by ∼80 Da (Figures 1F and S1B). Major peaks likely reflected persistent O-linked glycosylation not removed by PNGase F digestion. In TZM-bl neutralization assays, recombinant eCD4Ig-Emm06 demonstrated potent neutralization, with an 80% inhibitory concentration (IC80) of 167.2 ng/mL and a 14-fold lower *in vitro* half-maximal inhibitory concentration (IC50) compared with the previous eCD4-Ig variant (31.5 vs. 450.4 ng/mL) (Figures 1G and 4).^9^ Overall, these findings indicate that our HDAd vector produced a sulfated, stable, and highly potent eCD4-Ig therapeutic.

### Stable in vivo expression of CD4-Ig-Emm06 in murine models

**Figure 2:**
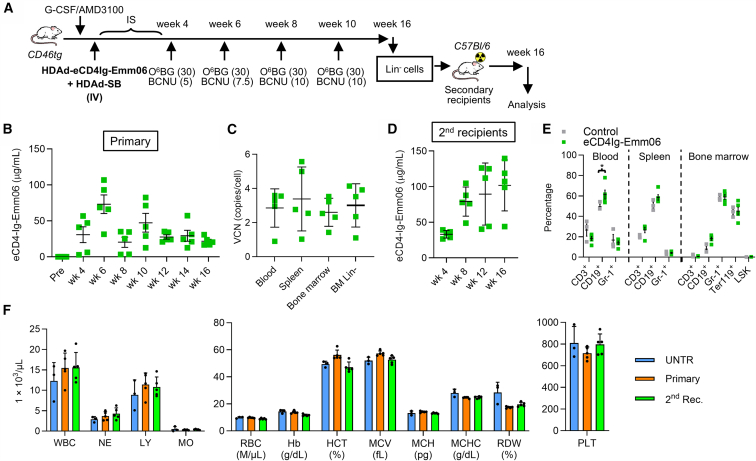
*In vivo* safety and efficacy studies in CD46-transgenic mice with HDAd-eCD4Ig-Emm06 Because DSG-transgenic mice do not adequately express the HDAd6/3+ receptor, DSG2, the eCD4Ig-Emm-6 viral genome was packaged into HDAd5/35++ capsids and tested in transgenic mice that express the HDAd6/35 receptor, CD46. (A) Schematic of the experiment. CD46-transgenic mice were mobilized with G-CSF/AMD3100 and, at the peak of mobilization, intravenously injected with HDAd5/35-eCD4Ig-Emm06 and HDAd5/35-SB at a 1:1 ratio (total dose: 1.6 × 10^10^ vp/kg). Mice were then subjected to four cycles of *in vivo* selection with O^6^BG and increasing doses of BCNU (5, 7.5, 10, 10 mg/kg). At week 16, *in vivo* transduced mice were sacrificed, and bone marrow lineage-negative (Lin^−^) cells were transplanted into lethally irradiated C57Bl/6 mice, which were followed for another 16 weeks (secondary recipients). IS, immunosuppression. (B) Serum eCD4Ig-Emm06 levels in *in vivo*-transduced mice. Each symbol represents an individual mouse. (C) Vector copy number (VCN) per cell in tissues of primary mice at week 16 after HDAd transduction. (D) Serum eCD4Ig-Emm06 levels measured in secondary mice, with each symbol representing an individual mouse. (E) Percentage of lineage-positive cells and HSCs in PBMCs, spleen, and bone marrow of primary *in vivo*-transduced mice at week 16. Shown are CD3^+^ (T cells), CD19^+^ (B cells) Gr-1^+^ (granulocytes), and Lin^−^/Sca^+^/c-Kit^+^ (LSK) cells. “Control” indicates control vector-transduced mice that underwent O^6^BG/BCNU selection. (F) Blood cell count analysis and erythroid parameters at week 16 of primary mice and secondary recipients compared with untransduced mice. Error bars denote SEM. Statistical comparisons for Figures 2E and 2F were performed using two-way ANOVA with Šídák’s multiple comparisons test. ∗*p* < 0.05, ∗∗*p* < 0.01, ∗∗∗*p* < 0.001, ∗∗∗∗*p* < 0.0001.

HDAd-eCD4Ig-Emm06 vectors were next evaluated in murine models to assess safety and transgene expression efficiency (Figure 2). Because DSG2-transgenic mice do not adequately express the HDAd 6/3+ receptor DSG2 on HSCs, HDAd-eCD4Ig-Emm06 genomes were instead packaged into HDAd5/35++ capsids for *in vivo* HSC transduction through CD46 in immunocompetent CD46-transgenic mice.^28^ Following HSC mobilization with G-CSF and AMD3100, HDAd-eCD4Ig-Emm06 and HDAd-SB vectors were infused intravenously into CD46-transgenic mice (*n* = 5) at 20 and 40 min after AMD3100 at 4 × 10^10^ virus particles per vector, with dexamethasone prophylaxis (Figure 2A).^9^ This was followed by four cycles of O^6^-benzylguanine/carmustine (O^6^BG/BCNU)-mediated selection.^29^ The treatment group was compared to non-transduced control mice (*n* = 3). In the treatment group, serum eCD4Ig-Emm06 levels exhibited an initial transient peak followed by a decline to stable concentrations, likely reflecting loss of non-integrated vector and persistence of integrated gene cassettes (Figure 2B). Serum levels stabilized at an average of 31.5 μg/mL (range 23.0–36.1 μg/mL), defined as the average from the final O^6^BG/BCNU treatment to necropsy. At necropsy of primary mice, the vector copy numbers (VCNs) were similar across the bone marrow, spleen, and blood and ranged from 0.9 to 5.6 copies/cell, indicating efficient transduction and durable persistence of modified HSCs (Figure 2C). Following transfer of HSCs into lethally irradiated secondary mice, eCD4Ig-Emm06 levels increased to an average 90.1 μg/mL (range 57.4–100.1 μg/mL), confirming sustained expression from engrafted modified HSCs (Figure 2D). HDAd infusion and selection were well tolerated. No cytopenias were observed relative to non-transduced controls, as assessed by flow cytometry in week 16 samples, including T cells (CD3^+^), B cells (CD19^+^), and granulocytes (Gr-1^+^) in blood, spleen, and bone marrow; erythroid cells (Ter119^+^) in bone marrow; and HSCs (Lin^−^/Sca^+^/c-Kit^+^ LSK cells) in the bone marrow of primary mice (Figure 2E). A small yet significant increase in CD19^+^ B cells was observed in the blood of HDAd-eCD4Ig-Emm06 animals compared with controls (*p* = 0.0346), but this did not reach significance in spleen or bone marrow (Figure 2E). Complete blood counts and white blood cell differentials at week 16 in primary and secondary mice further confirmed the absence of cytopenias across myeloid, lymphoid, and erythroid lineages (Figure 2F). Having established durable eCD4Ig-Emm06 expression and engraftment in mice, the HDAd platform for eCD4Ig-Emm06 delivery was next evaluated in the immunocompetent NHP model to assess efficacy, safety, and immunogenicity.

### Efficacy of HDAd-Delivered eCD4Ig-Emm06 in an NHP SIV challenge model

**Figure 3:**
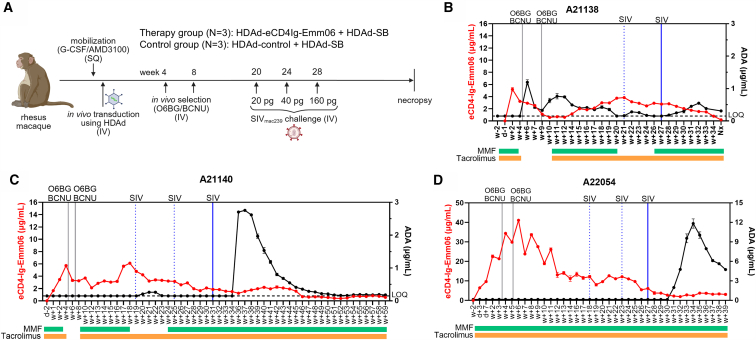
*In vivo* HSC transduction study in rhesus macaques (A) Schematic of the experiment created in https://BioRender.com. Animals were mobilized with G-CSF/AMD3100 and intravenously injected with either HDAd-eCD4Ig-Emm06 + HDAd-SB or HDAd-control + HDAd-SB. HSC mobilization involved two waves, with HDAd vectors intravenously injected at peak mobilization (8 h after AMD3100). The time of HAD injection is indicated as “0 h,” correspondingly at day −1 and day 0. Animals were pretreated with dexamethasone, tocilizumab, and anakinra. At weeks 4 and 8, O^6^BG/BCNU was administered for selection of modified cells. The first cycle of selection consisted of O^6^BG 120 mg/m^2^ and BCNU 10 mg/m^2^; the second cycle used the same O^6^BG dose, and BCNU was increased to 20 mg/m^2^. After at least 12 weeks from the final selection cycle, animals were challenged intravenously with SIVmac239 every 4 weeks using escalating doses until infection was established. (B–D) HDAd-eCDIg-Emm06 injected animals. Shown are the serum concentrations of eCD4Ig-Emm06 (red lines) and anti-eCD4Ig-Emm06 antibodies (anti-drug antibodies, ADA) (black line), measured in duplicate with SEM error bars. Treatment with immunosuppressants is indicated below the *x* axis: mycophenolate mofetil (MMF, green bars) and tacrolimus (orange bars). Vertical lines indicate O^6^BG/BCNU administration (black lines) and SIV challenges (blue lines). For SIV challenges, dashed blue lines represent challenges that did not result in infection, and solid blue lines indicate the challenge that established infection.

The HDAd 6/3+ platform for eCD4Ig-Emm06 delivery was tested in rhesus macaques allocated to eCD4Ig-Emm06 (*n* = 3) and control groups (*n* = 3) (Table S1; Figure 3A). All animals were confirmed to be seronegative to HDAd 6/3+ vectors at study entry. HDAd vectors were administered according to published protocols.^7^^,^^9^^,^^14^^,^^15^^,^^16^^,^^17^ Animals underwent HSC mobilization with G-CSF and AMD3100, followed by infusion of HDAd vectors on 2 consecutive days (1.5 × 10^12^ vp/kg/day). HSC mobilization was efficient, with peak CD34^+^/CD45RA^−^/CD90^+^ levels coinciding with HDAd infusions, confirming an optimized HDAd infusion regimen (Figure S2). Cytokine prophylaxis with dexamethasone, anakinra (IL-1 receptor antagonist), and tocilizumab (IL-6 receptor antagonist) effectively suppressed inflammatory responses: IL-6 levels rose transiently, peaking at 6 h post-HDAd injection and normalizing within 1–2 days (with no detectable elevation in A22065) (Figure S3). Notably, the highest IL-6 serum level in the study was ∼1,800 pg/mL, whereas IL-6 levels above 10,000 pg/mL are considered critical.^30^ All other tested cytokines (IL-2, IL-4, IL-5, TNF-α, and IFN-γ) remained below the limit of detection. Mild elevations in liver enzymes and thrombocytopenia were observed post-infusion and resolved within 1 week (Figure S4). Mobilization induced expected transient leukocytosis. HDAd infusion and O^6^BG/BCNU selection did not cause depletion of total leukocytes or leukocyte subsets (Figures S4–S7). Two cycles of O^6^BG/BCNU selection were performed, with additional cycles deferred due to BCNU-associated pneumonitis that occurred in 1 of 3 eCD4Ig-Emm06 animals and 2 of 3 control animals. Apart from BCNU-associated pneumonitis, the *in vivo* procedure was well tolerated, with no other clinically significant adverse events. No differences in toxicity were observed between eCD4Ig-Emm06 and control groups, supporting the safety of eCD4Ig-Emm06. Antibody responses against HDAd 6/3 capsids (IgM and IgG) were transient and not associated with clinical or laboratory abnormalities (Figure S8). Overall, while selection strategies for the HDAd platform require further optimization, HDAd gene therapy itself demonstrated a favorable safety profile, with substantially milder cytopenias than HSC transplantation-based approaches.^16^

### Quantification of serum eCD4Ig-Emm06

ELISAs were designed to measure eCD4Ig-Emm06 levels without cross-reactivity from endogenous anti-SIV antibodies that emerge after SIV infection. Various capture reagents were screened, including Env proteins from multiple HIV strains, the SIVmac239 Env protein, and anti-CD4 antibody clones known to cross-react with rhesus macaque CD4 (Figure S9A). To assess specificity, eCD4Ig-Emm06 was spiked into paired NHP serum samples collected before and after seroconversion. Previously reported ELISAs using SIVmac239 Env as the capture reagent cross-reacted with anti-SIV antibodies, limiting their utility for measuring eCD4-Ig after SIV infection.^2^^,^^9^ In contrast, using HIV BaL Env as the capture reagent provided superior sensitivity and specificity that was unaffected by the presence of anti-SIV antibodies. eCD4Ig-Emm06 levels in uninfected sera measured by the HIV BaL Env-based ELISA strongly correlated with those from the traditional SIVmac239 assay (Figure S9B, r = 0.9927, *p* < 0.0001), validating the HIV-based ELISA for quantifying eCD4Ig-Emm06 in serum.

**Figure 4:**
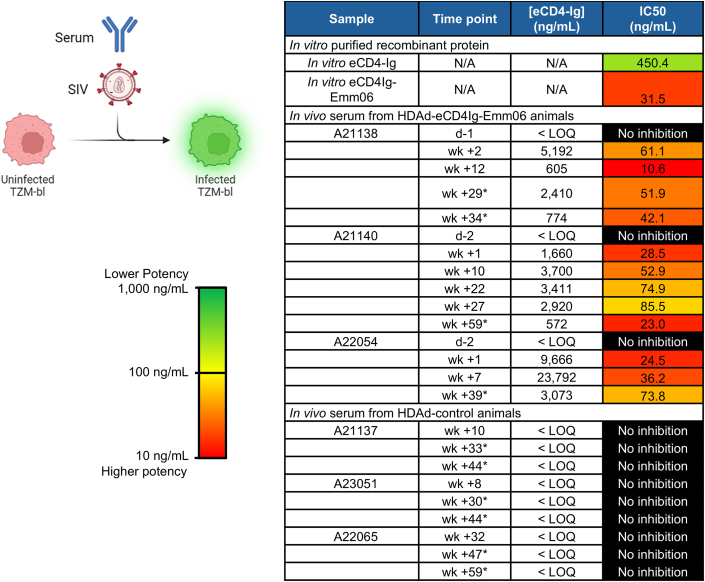
*In vitro* inhibition of SIV by serum eCD4Ig-Emm06 SIVmac239 was incubated in duplicate with TZM-bl cells and serial dilutions of recombinant eCD4-Ig or serum. Luciferase activity, as an indicator of SIV infection, was measured 2 days post-infection. IC50 was calculated as the eCD4Ig-Emm06 concentration or serum dilution required to reduce the maximum relative light units (RLU) by 50%. Asterisks (∗) indicate sera collected post-SIV infection. Schematic was created in https://BioRender.com.

The HIV-based ELISA was applied to measure serum concentrations of eCD4Ig-Emm06 (Figures 3B–3D and S10). Animals that received HDAd-eCD4Ig-Emm06 exhibited initial peaks in serum eCD4Ig-Emm06 levels that subsequently declined and stabilized, consistent with the loss of non-integrated vector genomes and the persistence of cells containing integrated cassettes, as confirmed by VCN measurements (Figures S11 and S12). eCD4-Ig levels stabilized between 2.0 and 20.6 μg/mL between the final O^6^BG/BCNU treatment and the first SIV challenge, which is 50- to 500-fold greater than the measured *in vitro* IC50 (Figure 4). *In vivo-*generated eCD4Ig-Emm06 was highly potent and exhibited neutralization efficacy comparable to *in vitro*-produced eCD4Ig-Emm06 (Figure 4), supporting the stability of the eCD4Ig-Emm06 variant and the efficacy of TPST2 co-expression in enhancing potency.^3^ In the control cohort, eCD4-Ig was undetectable except in one animal, in which transient levels were confirmed as false positives by ELISAs using a panel of SIV and HIV Env proteins (Figure S10D). Notably, all eCD4-Ig ELISAs were run with confirmatory ELISAs, and no other false positives were observed. Control sera lacked neutralizing activity (Figure 4), consistent with SIVmac239’s well-described resistance to neutralization.^31^

### Assessment of eCD4-Ig–Emm06 immunogenicity

To limit the potential for immune responses against eCD4Ig-Emm06 that pose a barrier to efficacy,^2^^,^^3^^,^^32^ animals were placed on immunosuppression with mycophenolate mofetil (MMF) and tacrolimus. This regimen successfully prevented the development of anti-eCD4Ig-Emm06 antibodies (Figures 3B–3D). Immunosuppression was briefly withheld during periods of BCNU-mediated pneumonitis for animals A21138 and A21140 (Figures 3B and 3C), during which anti-eCD4Ig-Emm06 antibodies emerged, with associated declines in eCD4Ig-Emm06 levels, likely due to accelerated elimination of eCD4Ig-Emm06 and its producer cells. Upon re-initiation of immunosuppression, anti-eCD4Ig-Emm06 antibodies declined. These findings suggest that the immunosuppression regimen of MMF and tacrolimus can effectively suppress anti-transgene immune responses during the uninfected phase. However, following SIV infection, all animals developed anti-eCD4Ig-Emm06 antibodies with subsequent declines in eCD4Ig-Emm06 levels, consistent with previous reports, and this demonstrates that MMF and tacrolimus are insufficient to suppress immunogenicity following SIV infection.^2^^,^^3^^,^^9^^,^^32^^,^^33^ In summary, our study uniquely evaluates the impact of immunosuppression on anti-eCD4-Ig immune responses and demonstrates that MMF and tacrolimus can suppress these responses during the uninfected phase but are insufficient during acute infection. Importantly, the presence of anti-eCD4Ig-Emm06 antibodies was not associated with reductions in neutralization potency of eCD4Ig-Emm06 (Figure 4), suggesting that anti-eCD4Ig-Emm06 antibodies did not impair eCD4Ig-Emm06 neutralization capacity. As noted earlier, histologic assessment of eCD4Ig-Emm06 and immune complexes was not possible due to the unavailability of specific detection antibodies in fixed tissues. While C3 levels can decline with immune complex disease, all animals demonstrated stable C3 levels (data not shown), which may support the absence of significant immune complex deposition, potentially mitigated by the IgG2 Fc domain in eCD4Ig-Emm06.^34^

Given that eCD4Ig-Emm06 expresses an affinity-enhanced CD4 domain, we evaluated whether the immunogenicity of eCD4Ig-Emm06 could be driven by binding to antigen-presenting cells (APCs) (Figure S13A). Compared with the parental rhesus eCD4-Ig, eCD4Ig-Emm06 exhibited increased binding to human (Raji, Ramos) and rhesus (LCL-8664) B cell lines, as well as the human monocyte cell line THP1 (Figure S13B). This enhanced binding was also observed in MHCII^−^ Raji cells (RJ2.2.5), indicating that the interaction between eCD4Ig-Emm06 and cells was not mediated by MHCII (Figures S13A and S13B). The CD32-blocking antibody FLI8.26 was used to assess the potential contribution of CD32 interactions to eCD4Ig-Emm06 binding (Figure S13C). FLI8.26 had no effect on the binding of eCD4Ig-Emm06 to rhesus LCL-8664 cells. Because significant eCD4Ig-Emm06 binding was observed to both human Raji cells and the MHCII^−^ subclone RJ2.2.25, CD32 involvement was further evaluated using RJ2.2.5 cells. Unexpectedly, FLI8.26 increased eCD4Ig-Emm06 binding (*p* = 0.0026), possibly by inducing a CD32 conformational change that enhanced binding. In contrast, FLI8.26 modestly reduced binding to human THP1 cells (*p* = 0.0027). Together, these results suggest that eCD4Ig-Emm06 binding is independent of MHCII, is partially mediated by CD32 on human monocytes, may involve CD32 on human B cells, and is not CD32 dependent on rhesus B cells.

### Biodistribution of *in vivo*-transduced cells

**Figure 5:**
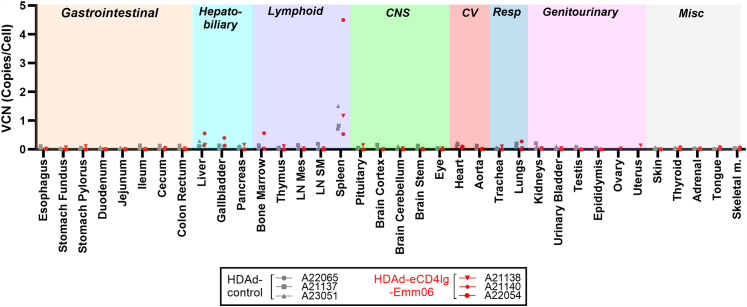
Tissue VCN Rhesus macaques treated with HDAd-eCD4Ig-Emm06 (*n* = 3, red) or HDAd-control (*n* = 3, gray) were infected with SIVmac239, and systemic tissues were analyzed at necropsy for VCN, measured by quantifying the *MGMT* transgene sequence. LN, lymph node; Mes, mesenteric; SM, submandibular. No statistically significant differences were observed between groups by two-way ANOVA with Šídák’s multiple comparisons correction.

**Figure 6:**
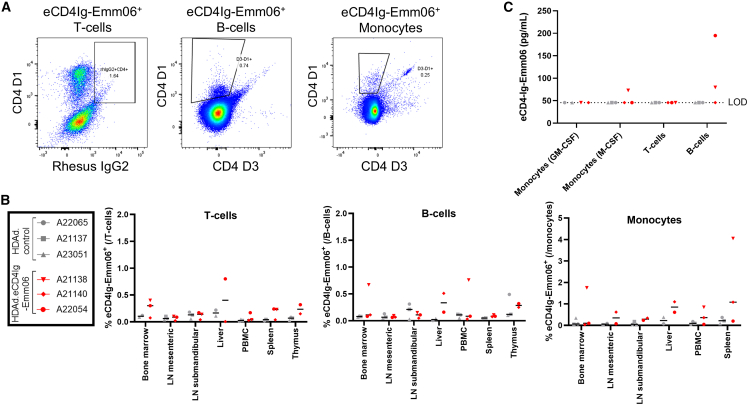
eCD4Ig-Emm06 expression from peripheral blood and tissue immune cells (A) Intracellular flow cytometry gating strategy to detect eCD4Ig-Emm06 expression from T cells, B cells, and monocytes from NHPs treated with HDAd-eCD4Ig-Emm06 or HDAd-control. Immune cell subsets were gated as previously described.^23^ Since eCD4Ig-Emm06 lacks a specific detection antibody, surrogate marker combinations were used. For CD3^+^CD20^-^ T cells, eCD4Ig-Emm06 expression was defined as cells co-expressing CD4 and IgG2, leveraging the fact that T cells naturally express CD4 but do not endogenously express IgG2. For CD3^−^CD20^+^ B cells, a subset of B cells naturally express IgG2, and CD4 can also be expressed via trogocytosis,^35^ so intracellular eCD4Ig-Emm06 was identified as cells that express the D1 domain but not the D3 domain of CD4, reflecting the D1 and D2-only structure of eCD4Ig-Emm06. CD11b^+^CD14^+^ monocytes can express CD4, and autofluorescence limits IgG2-based detection; therefore, eCD4Ig-Emm06 expression was identified by the presence of CD4 D1 and absence of CD4 D3. (B) eCD4Ig-Emm06 expression in T cell, B cell, and monocytes from peripheral blood and tissues at necropsy. Samples with <2,000 parent events were excluded. (C) Splenocytes were magnetic bead-purified into T cell, B cell, and monocyte subsets and cultured for 7 days *ex vivo*. Monocytes were differentiated with GM-CSF or M-CSF. eCD4Ig-Emm06 secretion was quantified by ELISA of cell culture supernatants. No statistically significant differences were observed between groups by two-way ANOVA with Šídák’s multiple comparisons correction.

The cellular and tissue distribution of eCD4Ig-Emm06 producing cells was mapped using VCN and protein expression analyses. VCN analyses confirmed that HDAd 6/3+ preferentially transduced HSCs *in vivo*, with peak levels of 0.1–1.2 copies/cell (Figures S11, S12, and S14). Among systemic tissues, the spleen exhibited the highest VCN (up to 4.5 copies/cell), indicating that this was the preferential homing site for transduced HSCs (Figure 5). No significant differences in VCN were observed between HDAD-eCD4Ig-Emm06 and HDAd-control groups, suggesting that eCD4Ig-Emm06 expression does not affect the survival of transduced cells. While O^6^BG/BCNU treatment did not significantly increase the VCN in peripheral blood mononuclear cells (PBMCs) and BM MNCs over time (Figures S11 and S12), these are minor compartments of transduced cells relative to the spleen (Figure 5), where selection would be expected to have the greatest impact but was not tested longitudinally in this study. Moreover, O^6^BG/BCNU selection was limited to two cycles due to BCNU-related toxicity, whereas we have previously observed that at least three cycles are necessary for effective enrichment.^9^ Transduced HSCs differentiated into lymphoid cells, with VCN in CD3^+^ T cell and CD20^+^ B cell subsets stabilizing to <0.05 copies/cell in PBMC and bone marrow (Figures S12 and S13). Next, protein expression of eCD4Ig-Emm06 was evaluated. Histologic assessment of eCD4Ig-Emm06 tissue distribution was unavailable due to the absence of specific detection reagents for fixed tissues. However, intracellular flow cytometry enabled detection of eCD4Ig-Emm06 within cell subsets. Using flow cytometry-based assays, eCD4Ig-Emm06 was detected in T cells, B cells, and monocytes, comprising <5% of each population across systemic tissues including bone marrow, lymph nodes, liver, PBMCs, spleen, and thymus (Figures 6A and 6B). Evaluation of the central nervous system indicated limited HDAd 6/3+ trafficking to this compartment, with VCN near the detection threshold (Figure 5), absence of eCD4Ig-Emm06 in cerebrospinal fluid, and no eCD4Ig-Emm06 protein expression observed in microglia. To assess the relative contribution of different cell subsets to eCD4Ig-Emm06 secretion, red blood cells (RBCs) were purified, and splenocytes were sorted into T cells, B cells, and monocytes. Each population was cultured *ex vivo*, and eCD4Ig-Emm06 levels were measured in the supernatant. No eCD4Ig-Emm06 production was detected from RBCs, as assessed by intracellular flow cytometry, cell lysis, and culture supernatants (Figure S15). Among splenocyte subsets, eCD4Ig-Emm06 production was detected from B cells in 2 of 3 animals (80–195 pg/mL supernatant) and from monocytes in 1 of 3 animals (73 pg/mL supernatant) (Figure 6C). No production was detected from T cells. One HDAd-eCD4Ig-Emm06 animal (A21140) lacked detectable eCD4Ig-Emm06 secretion during cell culture, likely due to incomplete spleen sampling and limited viability of thawed splenocytes. Overall, these findings demonstrate that HDAd-transduced HSCs gave rise to lymphoid and myeloid lineages that trafficked to systemic tissues.

### Protective efficacy of eCD4Ig-Emm06

**Figure 7:**
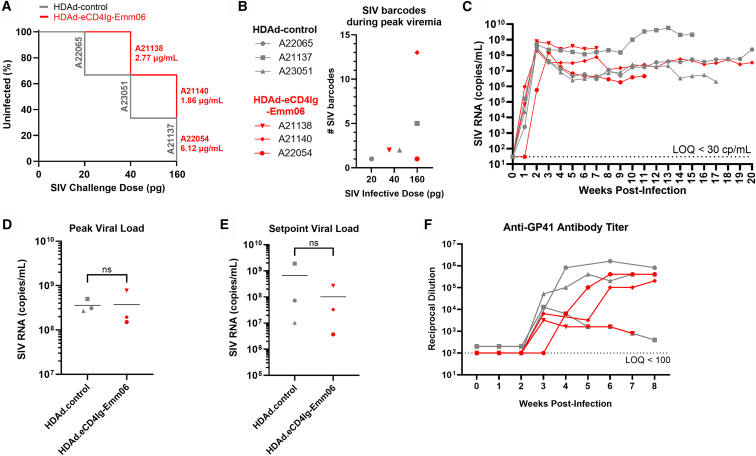
Viral load and survival curves Indian-origin rhesus macaques received either HDAd-eCD4Ig-Emm06 (*n* = 3, red) or HDAd-control (*n* = 3, gray) and were intravenously challenged every 4 weeks with escalating doses of SIVmac239 until infection was established. (A) Survival curve indicating the SIVmac239 dose at which infection occurred, as well as the corresponding animal ID and serum eCD4Ig-Emm06 level at the time of infection. No statistically significant differences were observed by the log-rank and Gehan-Breslow-Wilcoxon tests. (B) SIVmac239 barcodes measured during peak viremia to quantify founder viruses responsible for infection, with each barcode representing a distinct infectious event. (C) Longitudinal viral loads from the time of infection. (D) Peak and (E) setpoint viral loads compared between the HDAd-eCD4Ig-Emm06 and HDAd-control groups, with setpoint viral load defined as the average viral load after 5 weeks of infection. No statistically significant differences were observed by the unpaired *t* test. (F) Anti-SIV IgG responses measured by ELISA using an anti-gp41 format to avoid cross-reactivity with eCD4Ig-Emm06, which binds gp120.

**Figure 8:**
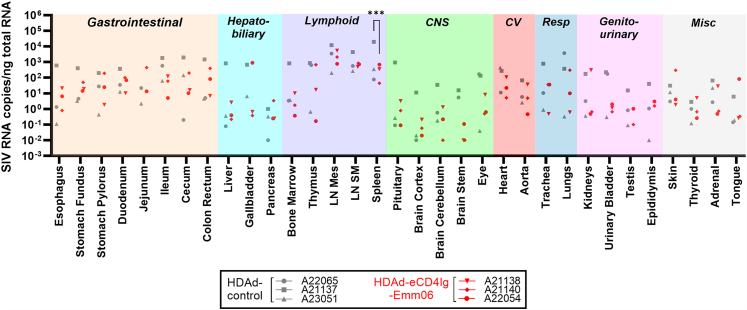
Tissue SIV viral loads Rhesus macaques were treated with HDAd-eCD4Ig-Emm06 (*n* = 3, red) or HDAd-control (*n* = 3, gray) and infected with SIVmac239. Systemic tissues were analyzed at necropsy for SIV viral loads, normalized to total RNA. HDAd-eCD4Ig-Emm06-treated animals showed significantly reduced SIV viral loads, as determined by two-way ANOVA with Šídák’s multiple comparisons correction (∗*p* < 0.05, ∗∗*p* < 0.01, ∗∗∗*p* < 0.001, ∗∗∗∗*p* < 0.0001). LN, lymph node; Mes, mesenteric; SM, submandibular.

The protective efficacy of *in vivo* eCD4Ig-Emm06 delivery was assessed by intravenously challenging animals with escalating doses of barcoded SIVmac239 (SIVmac239M2; 20 pg p27 = 13.5 TZM-bl TCID50 = 0.87 founders; see the materials and methods section)^36^ approximately every 4 weeks until infection was established. To allow for hematopoietic recovery, a minimum of 12 weeks elapsed between the final O^6^BG/BCNU dose and the first SIV challenge. Animals receiving HDAd-eCD4Ig-Emm06 became infected at similar SIVmac239 doses as controls (Figure 7A). Given the high pathogenicity of SIVmac239 and inter-animal variability that can limit the ability to measure protection in small cohorts, we harnessed molecular barcodes of the SIVmac239M2 challenge stock to quantify the number of viruses establishing infection (“founders”) as an additional measure of protection (Figure 7B).^36^^,^^37^ Founders were enumerated by measuring the number of unique barcodes during peak viremia, with each barcode representing a distinct infectious event. Higher SIVmac239 challenge doses were associated with expected increases in founder viruses.^36^ Notably, at the highest challenge dose of 160 pg, which corresponds to an average infectious dose of 7 founder viruses based on prior studies,^36^ a control animal was infected with 5 founders, an animal with low eCD4Ig-Emm06 concentrations (1.86 μg/mL) was infected with 13 founders, and the animal with the highest eCD4Ig-Emm06 concentrations (6.12 μg/mL) was infected with only a single founder virus. These findings suggest that higher eCD4Ig-Emm06 levels may block early infectious events. While no significant differences in viral loads were apparent at the cohort level between HDAd-eCD4Ig-Emm06 and HDAd-control groups (Figures 7C–7E), the animal with the highest serum eCD4Ig-Emm06 concentration (A22054) exhibited the lowest peak and setpoint viral loads, as well as a 1-week delay in viremia onset. These slower viral kinetics, despite challenge with the highest SIV dose, contrast with unprotected animals, which typically exhibit accelerated peak viremia at higher inocula.^36^ This animal also experienced the greatest recovery of CD4^+^CCR5^+^ T cells following early post-infection T cell depletion and sustained this subset through 10 weeks post-infection (Figure S16). The preferential protection of CCR5-expressing CD4^+^ T cells can be attributed to SIVmac239 being primarily CCR5-tropic and the mechanism of eCD4Ig-Emm06, which combines a CD4 domain and a coreceptor mimetic peptide to block SIV binding to the primary viral receptor CD4 and coreceptors such as CCR5 and CXCR4.^3^^,^^9^^,^^38^ Animals treated with HDAd-eCD4Ig-Emm06 had significantly lower viral loads in the spleen, which harbors the greatest concentration of transduced cells and thus likely the highest local concentrations of eCD4Ig-Emm06 (*p* = 0.0004) (Figure 8). While statistically significant, this comparison may have been disproportionately influenced by a control animal with higher splenic viral loads. Collectively, these findings suggest that HDAd-delivered eCD4Ig-Emm06 may suppress viral replication and slow disease progression, with greater benefits observed at higher eCD4Ig-Emm06 levels. Given the small sample sizes, these findings should be confirmed in larger cohorts.

Immunologic factors may have contributed to the incomplete protection observed. Although controller MHC alleles were balanced between the control and eCD4Ig-Emm06 cohorts, the control animal with the lowest SIV viral loads (A23051) had the greatest number of controller alleles (Table S2). Additionally, the two animals with the highest SIV viral loads (A22065 and A21138) had the lowest anti-SIV antibody titers, measured by an anti-gp41 ELISA designed to avoid cross-reactivity with eCD4Ig-Emm06 (Figure 7F). This raises the possibility that O^6^BG/BCNU selection may have impaired endogenous immune responses and limited the therapeutic benefit of eCD4Ig-Emm06. Non-genotoxic selection strategies may better preserve endogenous immunity and improve therapeutic outcomes.^39^

### Evaluation for viral escape

Although eCD4-Ig possesses exceptional breadth and a high barrier to viral resistance, viral escape can occur at a significant fitness cost.^2^^,^^3^^,^^5^^,^^32^ To evaluate viral escape, long-read sequencing of the Env region was performed on plasma SIVmac239 virus collected 11 week post-infection (Figures S17–S22), with the exception of A21138, which was sequenced at 7 weeks post-infection due to early necropsy for SIV-associated thrombocytopenia.^40^ Comparisons between the control and eCD4Ig-Emm06 cohorts focused on mutations that (1) comprised at least 10% of reads, (2) occurred within eCD4Ig-Emm06 binding sites (V2 apex, V3 base, V4, and the CD4 binding site),^5^ and (3) were exclusive to the eCD4Ig-Emm06 cohort to exclude mutations arising from natural evolution or endogenous humoral immune pressure.^41^^,^^42^ Under these criteria, no Env mutations were enriched in the eCD4Ig-Emm06 cohort, and no previously described eCD4-Ig escape mutations were observed in either cohort, suggesting the absence of viral escape in this study.^2^

## Discussion

In our NHP model of gene therapy and HIV infection, we demonstrate that the HDAd 6/3+ platform is a promising *in vivo* gene therapy approach for delivering biologic therapeutics. We achieved stable, long-term expression of a sulfated, affinity-enhanced variant of eCD4-Ig (“eCD4Ig-Emm06”) that retained *in vivo* neutralization efficacy. Transduced HSCs differentiated into both lymphoid and myeloid lineages and trafficked to systemic tissues, with B cells emerging as the principal source of eCD4-Ig production. HDAd delivery of eCD4Ig-Emm06 suggested reduced splenic viral reservoirs, and the highest levels resulted in fewer founder SIV viruses establishing infection, delayed onset of viremia, lower plasma viral loads, and protection against CD4^+^CCR5^+^ T cell depletion, although these findings should be confirmed in larger studies given the small sample size. We identify future strategies to improve efficacy by leveraging lineage-specific expression, minimizing immunogenicity, and enhancing selection of edited cells.

We previously demonstrated the efficacy of the HDAd 5/35++ platform to deliver eCD4-Ig, which combines the Ad5 capsid with an affinity-modified Ad35 fiber knob for enhanced binding to CD46 on HSCs.^7^^,^^9^ While effective, this platform is limited by pre-existing immunity against Ad5 in up to >90% of humans.^18^ To address this, we substituted the capsid with Ad6, a rare serotype with <10% seroprevalence in humans^18^ that also offers a favorable safety profile, with reduced cytokine release and decreased off-target transduction of hepatocytes.^6^ Additionally, binding of the Ad35++ fiber knob to CD46 on NHP erythrocytes can reduce transduction efficiency and limit efficacy evaluation in NHP models.^7^^,^^14^ Therefore, we replaced the Ad35++ fiber knob with the Ad3 fiber knob, which targets DSG2 for specific HSC transduction in both NHP and human cells.^14^^,^^19^ CXCR4 was also transiently expressed to enhance homing of transduced HSCs to the bone marrow.^14^ Together, these modifications define our HDAd 6/3+ platform, which has been optimized for enhanced translational potential. In this study, we show that the HDAd 6/3+ platform achieves modestly improved transduction efficiencies with reduced toxicity in HSCs, supporting its further development.

We implemented HDAd 6/3+ vectors to transduce HSCs *in vivo* in rhesus macaques to express eCD4-Ig, a secreted decoy receptor composed of a CD4 domain linked via an Fc domain to a coreceptor mimetic peptide that binds to conserved regions of the HIV envelope.^2^^,^^3^^,^^5^ To reduce immunogenicity and support sustained eCD4-Ig levels, we used a rhesus-adapted version (rh-eCD4-Ig) that incorporates an IgG2 Fc domain.^9^^,^^33^ We further enhanced rh-eCD4-Ig by introducing the LS mutation and space-filling mutations to generate the eCD4Ig-Emm06 variant, which exhibited a 14-fold increase in potency over the parental protein.^24^^,^^25^^,^^26^ In our HDAd 6/3+ vectors, eCD4Ig-Emm06 was expressed under the constitutive EF1α promoter within an integrated cassette that also encoded the sulfation enzyme TPST2, which is necessary to sulfate tyrosine residues in eCD4Ig-Emm06 for efficient HIV neutralization.

Rhesus macaques were assigned to receive either an HDAd 6/3+ vector expressing eCD4Ig-Emm06 (*n* = 3) or a control HDAd vector (*n* = 3). Post-infusion laboratory abnormalities were brief, including transient mild elevations in IL-6 that normalized within 1–2 days, and elevated liver enzymes and thrombocytopenia that resolved within 1 week. O^6^BG/BCNU selection of MGMT^P140K^-expressing transduced cells was limited to two cycles due to BCNU-related pneumonitis, which precluded additional cycles necessary for effective enrichment.^9^ Bone marrow aspirates revealed preferential HSC transduction, confirming the specificity of the HDAd 6/3+ platform. The highest VCN was observed in the spleen (up to 4.5 copies/cell), suggesting that despite our previous observation that transient CXCR4 expression enhances bone marrow homing, most transduced HSCs continue to home to the spleen.^14^ Although these splenic HSCs are functional, optimal outcomes would be achieved if HSCs homed back to the bone marrow, the primary site of hematopoiesis, to maximize transgene-expressing cells.^14^^,^^43^ Future strategies to improve bone marrow homing could target additional mechanisms, such as VLA-4 expression.^44^ VCN and flow cytometry analyses demonstrated that gene-modified HSCs differentiated into lymphoid and myeloid progeny cells, including T cells, B cells, and monocytes, which trafficked to the periphery, lymph nodes, and liver. Importantly, *ex vivo* cell cultures of purified cell populations identified B cells as the principal eCD4Ig-Emm06 producers. Correspondingly, serum eCD4Ig-Emm06 levels were stably maintained at concentrations 50- to 500-fold above the IC50.

Our experience with eCD4-Ig delivery using HDAd 6/3+ vectors in this study and HDAd 5/35++ vectors in our prior work highlights key differences,^9^ although comparisons should be interpreted cautiously because HDAd 6/3+ vectors encoded a different eCD4-Ig variant with additional TPST2 expression. While HDAd 6/3+ vectors achieved ∼10-fold higher initial PBMC gene marking, BCNU-associated pneumonitis limited the number of O^6^BG/BCNU selection cycles with this vector system. By contrast, BCNU selection has been well tolerated with HDAd 5/35++ vectors,^20^^,^^45^^,^^46^^,^^47^^,^^48^ allowing more selection cycles to achieve greater enrichment of transduced cells and higher long-term eCD4-Ig levels. These findings support exploring alternative selection strategies for the HDAd 6/3+ platform in future studies. Both vectors demonstrated modest inter-animal variability in transduction efficiency and eCD4-Ig levels, likely reflecting host-specific differences in HSC mobilization and immune responses, despite weight-based dosing of the mobilization regimen, vector, and immunosuppression.^49^^,^^50^ Ongoing platform refinements aim to reduce this variability and enable fine-tuning of therapeutic output through improved selection of transduced cells, as discussed below. Both vectors induced transient IL-6 elevations, whereas HDAd 5/35++ vectors also caused a transient TNF-α increase (∼50 pg/mL) of unclear significance in the absence of overt inflammatory toxicity. Overall, both platforms enable HSC-specific transduction and durable therapeutic delivery, and vector choice should be guided by pre-existing immunity and compatible selection strategies.

This study provides evidence in a NHP model that HSC gene therapy can generate modified B cells for therapeutic delivery. This approach creates opportunities for sustained *in vivo* production of HIV therapeutics, including eCD4-Ig, bNAbs, and HIV fusion inhibitors. HSC-derived B cells offer several advantages for therapeutic delivery. While direct modification of B cells can be limited by short durations of antibody production,^51^ HSC-derived B cells offer a durable source of engineered B cells that support long-term therapeutic production. A unique feature of HSC-based gene therapies is the ability to select for transduced cells to boost therapeutic output.^20^^,^^45^^,^^46^^,^^47^^,^^48^ In addition to BCNU selection, several alternative selection platforms are under investigation, including tHMGA2-driven auto-expansion of transduced cells.^39^ Signaling-capable eCD4-Ig or bNAb constructs may also enable selection through immunization or viral replication to drive antigen-dependent clonal expansion and somatic hypermutation for enhanced eCD4-Ig expression and binding affinity.^51^^,^^52^^,^^53^ HSC-mediated delivery also enables therapeutic production from erythroid lineages. Erythropoiesis accounts for nearly a quarter of cells in the body and contributes enormous protein biosynthesis, as exemplified by hemoglobin production.^54^ While we have previously used HDAd vectors to drive erythroid expression of bioengineered factor VIII for hemophilia A,^55^ we did not observe eCD4-Ig production from RBCs in the current study, potentially due to the absence of the β-globin 3′UTR. Incorporating a β-globin 3′UTR in future vectors may stabilize transgene mRNA to enable robust eCD4-Ig production from erythroid cells.^55^

To evaluate the efficacy of HDAd-delivered eCD4Ig-Emm06, animals underwent serial intravenous escalating-dose SIVmac239 challenges to model protection against HIV infection. Although HDAd-eCD4Ig-Emm06 animals became infected with SIVmac239 at similar doses as controls, SIV barcode analyses demonstrated that this infection model was highly stringent, with the highest challenge dose resulting in up to 13 founder viruses—far exceeding the one founder virus typically observed in sexual HIV-1 transmission and the median three viruses seen with intravenous transmission.^36^^,^^56^^,^^57^ Barcode analyses enabled high-resolution assessment of protective efficacy and demonstrated that the highest eCD4Ig-Emm06 levels (∼6 μg/mL) effectively reduced the number of founder SIV viruses, indicating blockade of critical early infection events. The protective effect of eCD4Ig-Emm06 was further confirmed by reduced SIV viral loads and slowed disease progression post-infection. The animal with the highest eCD4Ig-Emm06 levels demonstrated delayed onset of viremia, lower peak and setpoint viral loads, and improved recovery of CD4^+^CCR5^+^ T cells. HDAd-eCD4Ig-Emm06 animals also exhibited significantly reduced splenic viral reservoirs, likely due to higher local concentrations of eCD4Ig-Emm06, as this site contained the highest concentration of transduced cells (VCN up to 4.5 copies/cell). These virologic benefits are compelling, given their divergence from the reproducible and consistent SIVmac239 infection dynamics in unprotected animals.^36^ The promising results of this proof-of-concept study warrant confirmation in larger-scale studies.

Similar to broadly neutralizing antibodies, higher eCD4Ig-Emm06 levels may afford greater protection, as suggested by the favorable viral kinetics observed in the study animal with the highest eCD4Ig-Emm06 levels.^58^ Accordingly, efficacy may improve by increasing eCD4Ig-Emm06 levels using augmented HSC gene therapy approaches featuring lineage-specific expression and enhanced selection, as discussed above. Overcoming eCD4Ig-Emm06 immunogenicity would also assist in achieving high therapeutic levels by reducing clearance of eCD4Ig-Emm06 and its producer cells. The primary source of immunogenicity has been well characterized as the coreceptor mimetic peptide, with a minor contribution from an epitope formed by the rhesus CD4^I39N^ and Fc domains.^2^^,^^3^^,^^32^^,^^33^ To mitigate immunogenicity, we used a less immunogenic IgG2-Fc domain^2^^,^^3^^,^^33^ and applied immunosuppression with MMF and tacrolimus as a proof-of-concept to suppress immunogenicity and assess whether subsequent tapering could permit sustained tolerance. Our study demonstrated that immunosuppression effectively suppressed anti-eCD4Ig-Emm06 antibody responses. However, these responses emerged upon withdrawal of immunosuppression, accompanied by declines in eCD4Ig-Emm06 levels. In addition, all animals developed anti-eCD4Ig-Emm06 antibodies following SIV infection despite immunosuppression, consistent with prior reports.^2^^,^^3^^,^^32^^,^^33^ The mechanisms underlying enhanced immunogenicity post-SIV infection remain undefined but may involve immune complex formation between SIV and eCD4Ig-Emm06,^59^^,^^60^^,^^61^ as well as generalized immune activation during productive infection.^62^ We also observed that eCD4Ig-Emm06 bound non-specifically to cells. Human versions of eCD4-Ig under development are more optimized, less prone to aggregation than the available rhesus versions, such as eCD4Ig-Emm06, and less immunogenic coreceptor mimetic peptides are also being developed. Finally, efficacy may be further enhanced through combination approaches, such as co-delivery of multiple neutralizing therapeutics.^63^ For example, combining eCD4-Ig with V3-targeting bNAbs may synergistically improve antiviral activity.^5^ The HDAd platform possesses a large cargo capacity (∼35 kb), making it well suited for delivering multiple transgenes for such combination therapeutic strategies.

In summary, this proof-of-concept study demonstrates that *in vivo* HSC transduction using the HDAd 6/3+ platform enables durable expression of a potent, sulfated eCD4-Ig variant capable of reducing splenic viral reservoirs in an NHP challenge model with SIVmac239. B cells emerged as a major source of eCD4Ig-Emm06, highlighting the potential to leverage B-lineage cells for sustained therapeutic antibody delivery and vaccine-like applications. The animal with the highest circulating eCD4Ig-Emm06 levels exhibited fewer founder viruses establishing infection, delayed onset of viremia, lower viral loads, and protection of CD4^+^CCR5^+^ T cells, which are promising findings that warrant confirmation in larger studies. Strategies to further enhance eCD4-Ig expression include the use of less immunogenic eCD4-Ig variants, combination bone marrow homing strategies, and improved selection methods. These results lay a foundation for advancing HSC-based gene therapy for durable delivery of broadly neutralizing biotherapeutics, with broad applicability to complex infectious diseases.

## Materials and methods

### HDAd vectors

The construction of the Ad6/3+ helper vector is described elsewhere.^64^ For the production of HDAd6/3+ vectors, the corresponding plasmids were linearized with *PmeI* and rescued in 116 cells using Ad6/3 helper virus. The SB100× expressing vectors were generated previously.^8^ HDAd vectors were produced in 116 cells as described in detail elsewhere.^65^ Helper virus contamination levels in the final preparations were <0.05%. Titers ranged 9–12 × 10^12^ virus particles(vp)/ml. HDAd-GFP vectors showed an approximate vp:transduction unit ratio of 20:1 in five independent preparations. Viruses were stored in 10 mM Tris, pH 7.5, 10 mM MgCl_2_, and 10% glycerol at −80°C. Before injection into NHPs, HDAds were dialyzed against 25 mM Tris, pH 7.5, 140 mM NaCl, 5 mM KCl, 0.6 mM Na2HPO4, 0.5 mM MgCl2, 0.9 mM CaCl2, and 5% sucrose.^7^

### *In vitro* HSC transduction

*In vitro* transduction was performed as previously described.^64^ Human CD34^+^ cells from mobilized healthy donors were obtained from the Fred Hutchinson Cancer Center, Seattle, Washington. NHP CD34+ cells were used fresh after magnetic cell separation with the CD34 MicroBead kit (Miltenyi). Cells were incubated in serum-free medium (Stemspan SFEMII, Stemcell Technologies) supplemented with penicillin/streptomycin (Gibco), Flt3 ligand (Flt3-L, 100 ng/mL), thrombopoietin (TPO, 100 ng/mL), and stem cell factor (SCF, 100 ng/mL), as well as the small molecules StemRegenin1 (SR1, 1 μΜ) (Cellagen Technology) and Ly2228820 (Ly, 100 nM) (Selleckchem). All cytokines were obtained from PeproTech. CD34^+^ cells were transduced with the HDAd vectors in low-attachment plates for 48 h and then analyzed by flow cytometry.

### eCD4Ig-Emm06 generation

293T cells were seeded in 150 mm cell culture plates and transduced with HDAd-eCD4Ig-Emm06 at an MOI of 1,000 vp/cell. Supernatant was harvested 48 h later, and eCD4Ig-Emm06 was purified using a Protein A Sepharose column (Genscript). Eluted fractions confirmed to contain eCD4Ig-Emm06 by SDS-PAGE under reducing conditions were pooled and dialyzed against PBS (pH 7.4). The product was then concentrated using a 10 kDa MWCO concentrator (Amicon), adjusted to 1× PBS with 5% glycerol, and stored at −80°C.

### Intact protein mass spectrometry

Samples were deglycosylated using 2 μL (500 unit/uL) of PNGase F for every 20 μL of sample and incubated at 37°C, 400 rpm, for 30 h. Next, the buffer was exchanged seven times with 200 mmol/L ammonium acetate buffer (pH 7; 99.99%, Millipore Sigma) using Amicon Ultra-0.5 mL centrifugal filters with a 10 kDa molecular weight cutoff (Millipore Sigma) at 14,000 × g. The protein concentration of the final solution was measured using a NanoDrop. Four microliters of the sample were loaded into a platinum-coated emitter and electrosprayed directly into an Exploris 480 Orbitrap mass spectrometer (Thermo Fisher Scientific) with the Biopharma extended mass range option. Instrument settings were as follows: capillary voltage, 1.5 kV; capillary temperature, 100°C; in-source collision-induced dissociation (CID), 15–135 eV; and higher-energy collisional dissociation (HCD) collision energy, 5–20 V. Mass spectra were deconvoluted and processed using UniDec software.

### Sex as a biological variable

Our study examined both male and female mice and rhesus macaques. Although sex was not balanced among control and treatment groups due to animal availability, similar findings are reported in both sexes.

### Animals

All experiments involving animals were conducted in accordance with the institutional guidelines set forth by the University of Washington and under conditions that meet NIH standards, as stated in the *Guide for the Care and Use of Laboratory Animals* (National Research Council, National Academy Press, Washington, DC, 1996), ILAR recommendations, the American Association for Accreditation of Laboratory Animal Care (AAALAC) requirements, Office of Laboratory Animal Welfare (OLAW) Public Health Service (PHS) policy, USDA Animal Welfare Act and Regulations, and the University of Washington’s Institutional Animal Care and Use Committee (IACUC) policies. The studies were approved by the University of Washington IACUC (protocol no. 3108-01 for mice and 3108-04 for NHPs). Mice were housed in specific-pathogen-free facilities, and NHPs were housed at the Washington National Primate Research Center (WaNPRC). All experiments adhered to Animal Research: Reporting of *In Vivo* Experiments (ARRIVE) guidelines and NIH recommendations on experimental design and reporting standards.

### Mice

hCD46-transgenic mice are C57Bl/6-based transgenic mice that contain the human CD46 transgene.^66^ For HSC mobilization and *in vivo* transduction, HSCs were mobilized in mice by subcutaneous (s.c.) injections of human G-CSF (Amgen) 5 μg/day for 4 days, followed by s.c. AMD3100 at 5 mg/kg on day 5. Cytokine prophylaxis consisted of intraperitoneal (i.p.) dexamethasone (Fresenius Kabi) at 10 mg/kg, administered 16 h and 2 h prior to HDAd injection. HDAd vectors (4 × 10^10^ vp/dose) were injected into the retro-orbital plexus at 30 and 60 min after AMD3100. *In vivo* selection was performed every 4 weeks for four cycles. Each cycle consisted of two i.p. doses of O^6^BG at 15 mg/kg, given 30 min apart. BCNU was administered via the i.p. route at 5 mg/kg during the first cycle, 7.5 mg/kg in the second cycle, and 10 mg/kg in the third and fourth cycles. For secondary BM transplantation, C57BL/6J mice that were 6–8 weeks old (Jackson Laboratory) were used as secondary recipients. Bone marrow HSCs were harvested from primary mice at 16 weeks post-transduction, and HSCs were enriched using the CD34 Microbead Kit (Miltenyi). Secondary recipient mice were irradiated with 1,000 Rad and injected with 1 × 10^6^ HSCs per mouse. Secondary recipients were followed for 16 weeks post-transplantation before terminal analyses.

### NHP

Six Indian-origin rhesus macaques (*Macaca mulatta*) were housed at the Washington National Primate Research Center (WaNPRC). All animals were SIV negative and seronegative for HDAd 6/3+ vectors at study entry. At study initiation, average body weights were 5.3 kg for the control group and 3.9 kg for the eCD4Ig-Emm06 group; average ages were 4.2 and 3.0 years, respectively. Haplotypes associated with SIV control were balanced between groups.^67^ MHC genotyping was performed using Fluidigm Access Array exon 2 amplification from genomic DNA templates isolated from PBMCs and whole blood samples, followed by Illumina MiSeq sequencing.^68^

HSCs were mobilized using s.c. G-CSF (Amgen) at 50 μg/kg each morning from day −5 to day 0 and s.c. AMD3100 (Calbiochem) at 5 mg/kg at midnight on day 0 (8 h prior to HDAd dosing). Cytokine prophylaxis included intravenous (i.v.) dexamethasone (Fresenius Kabi) at 4 mg/kg, administered at 2:00 p.m. on day −1 and at 7:30 a.m. and 2:30 p.m. on day 0. Anakinra/Kineret (Swedish Orphan Biovitrum) was administered s.c. at 50 mg/dose on day −1 and on day 0 (1 h before and 6 h after HDAd injection). Tocilizumab/Actemra (Genentech) was given i.v. at 8 mg/kg on both day −1 and day 0 (1 h before and 6 h after HDAd injection). HDAd vectors were infused at 1.5 × 10^12^ vp/kg on days −1 and 0. *In vivo* selection was performed with O^6^BG (120 mg/m^2^) and BCNU (10 mg/m^2^) administered approximately 4 weeks after HDAd infusion, followed by a second dose of O^6^BG (120 mg/m^2^) and BCNU (20 mg/m^2^) at approximately 8 weeks. After at least 12 weeks had elapsed since the final O^6^BG/BCNU dose, NHPs were intravenously challenged with barcoded SIVmac239M2^36^ approximately every 4 weeks using serial escalating doses until infection was established, followed by terminal analyses approximately 12 weeks post-infection. SIVmac239M2 virus stocks were previously described,^36^ and titers were determined to be 100,848 pg p27/mL and 68,000 TZM-bl TCID_50_/mL using published protocols.^36^ The founder titer was measured by quantifying unique barcodes 1 week post-infection in a preliminary cohort of intravenously infected rhesus macaques, yielding a founder titer of 4,389 founders/mL of virus stock. Based on these measurements, SIVmac239M2 titers correspond to 20 pg p27 = 13.5 TCID_50_ = 0.87 founders.

### Tissue processing to single-cell suspensions

Lymph nodes, spleen, and liver tissues were mechanically dissociated by mincing and filtering through a 70 μm filter. Gastrointestinal, lung, and thymus tissue were minced and incubated with 8 μg/mL Liberase (Roche) and 40 μg/mL DNAse I (Sigma) for 30–60 min at 37°C with vigorous stirring, followed by straining through a 70 μm filter. Splenic and lung tissues also underwent RBC lysis using ACK lysing buffer (Life Technologies). Brain tissue was minced, transferred to a douncer, and sheared using four strokes. The brain tissue was resuspended in 30% Percoll (VWR) and centrifuged at 3,000 × g for 10 min at 4°C, without a brake. The myelin layer was aspirated, and the microglia cell pellet was resuspended in PBS and centrifuged at 1,000 × g for 5 min. Microglia underwent hemolysis using ACK lysing buffer, followed by CD11b bead selection (Miltenyi) according to manufacturer protocols. Bone marrow aspirates underwent RBC lysis using hemolytic buffer (150 mM ammonium chloride [Thermo Fisher Scientific], 12 mM sodium bicarbonate [Sigma-Aldrich], 0.1 mM EDTA [Invitrogen]) before downstream assays. Cells were cryopreserved in CryoStor CS10 (StemCell) and stored in liquid nitrogen.

### Plasma SIV viral load

Viral RNA was isolated from plasma using the MasterPure RNA Purification Kit (Lucigen) according to the manufacturer’s instructions. Precipitated RNA and RNA standards were amplified using SIV gag-specific primers (forward: 5′-ACT TTC GGT CTT AGC TCC ATT AGT G-3′; reverse: 5′-TTT TGC TTC CTC AGT GTG TTT CA-3′) and probe (5′-TTC TCT TCT GCG TGA ATG CAC CAG ATG A-3′). A two-step quantitative reverse-transcription PCR (RT-qPCR) was performed on a QuantStudio 3 system (Thermo Fisher Scientific).

### Nucleic acid isolation from tissues

Tissues were homogenized in RNA*Later* Solution (Thermo Fisher Scientific) using the Precellys 24 homogenizer (Bertin). Genomic DNA was isolated using the PureLink Genomic DNA Mini Kit (Thermo Fisher Scientific), and cellular RNA was isolated using the NucleoSpin RNA Mini kit (Takara) according to the manufacturer instructions.

### Tissue SIV viral load

Cell-associated RNA was isolated from tissues using the protocols described above and added to 50 μL of guanidine hydrochloride with proteinase K (3 M guanidine hydrochloride [Sigma-Aldrich], 50 mM Tris·HCl [pH 7.6], 1 mM CaCl_2_, 1 mg/mL proteinase K [Thermo Fisher Scientific]). This mixture was incubated at 37°C for 1 h, followed by the addition of 200 μL of guanidine thiocyanate with glycogen (5.7 M guanidine thiocyanate [Sigma-Aldrich], 50 mM Tris·HCl [pH 7.6], 1 mM EDTA [Invitrogen], 600 μg/mL glycogen [Roche]). Samples were then incubated at room temperature for 5 min. Next, 250 μL of 100% isopropanol was added, and nucleic acid was precipitated by centrifugation at 20,000 × g for 10 min. The pellet was washed with 70% ethanol and centrifuged at 20,000 × g for 10 min. Pellets were allowed to air dry and then dissolved in 30 μL Qiagen AVE buffer.

One-step RT PCR was performed on the Applied Biosystems Quantstudio 7 Flex using PCR volumes of 30 μL, composed of 10 μL of RNA, Qiagen Quantitect virus kit, 900 nM forward and reverse primers (forward 5′-GCA GAG GAG GAA ATT ACC CAG TAC-3′, reverse 5′-CAA TTT TAC CCA GGC ATT TAA TGT T-3′), and 200 nM probe (5′-TGT CCA CCT GCC ATT AAG CCC GA-3′). A standard curve was constructed using gag RNA transcripts quantitated by ddPCR. All samples and standards were run in duplicate. Reverse transcription was performed at 50°C for 20 min, followed by a hot start at 95°C for 5 min, and qPCR cycling parameters of 95°C for 20 s and 60°C for 45 s for 45 cycles of amplification. No-RT PCR reactions were included to detect contaminating DNA.

### VCN

The pHCA-EF1α-eCD4-Ig plasmid was serially diluted to generate a standard curve. VCN was quantified using a StepOnePlus real-time PCR system (Applied Biosystems) with Power SYBR Green PCR Master Mix and primers targeting the transgenic rhesus mgmt sequence (forward: 5′-GCT GTC TGG TTG TGA GCA GGG TCT-3′; reverse: 5′-CGG GCT GAT GGA AAT AGG CAT TC-3′). Reactions were performed with 10 ng of genomic DNA (∼1520 cells) in 20 μL volumes and measured in triplicate.

### SIV viral sequencing

Viral RNA was isolated from serum samples using the QIAamp MinElute Virus Spin Kit or QIAamp Viral RNA Mini Kit (Qiagen), following the manufacturer’s protocol. For sequencing the Env region, reverse transcription of purified RNA was performed using the SuperScript IV First-Strand Synthesis System (Thermo Fisher Scientific) and the reverse primer 5′-CTC TCT CTT CAG CTG GGT-3′ with an additional adaptor sequence and a 4-nucleotide index sequence for multiplexing. Residual primers and deoxyribonucleotide triphosphates (dNTPs) were removed using ExoSAP-IT PCR Product Cleanup Reagent (Applied Biosystems). The resulting complementary DNA (cDNA) was amplified by PCR using the KAPA HiFi HotStart ReadyMix PCR Kit (Roche), forward primer 5′-GTA GAT GTC TAG GGG AAG GAC-3′, and reverse primer targeting the adaptor sequence. PCR products were purified using Agencourt AMPure XP beads (Beckman Coulter), and excess primers and dNTPs were removed again with ExoSAP-IT. DNA quality and fragment size distribution were evaluated using the Agilent Genomic DNA ScreenTape System, and concentrations were determined via Qubit fluorometric quantification (Thermo Fisher Scientific). Purified amplicons were submitted to Quintara for long-read sequencing. Sequence data files were processed using an in-house deep sequencing analysis pipeline that has been uploaded to Github. Sequences with early stop codons and large deletions (>10 amino acids) were excluded from analyses.

For barcode sequencing, cDNA was generated using Superscript IV reverse transcriptase (Invitrogen) and an SIV-specific reverse primer (5′-CAG GTT GGC CGA TTC TGG AGT GGA TGC-3′). The cDNA was quantified via qRT-PCR using the primers VpxF1 (5′-CTA GGG GAA GGA CAT GGG GCA GG-3′) and VprR1 (5′-CCA GAA CCT CCA CTA CCC ATT CATC-3′) with a labeled probe (5′-ACC TCC AGA AAA TGA AGG ACC ACA AAG GG-3′). PCR was performed using High-Fidelity Platinum Taq (Thermo Fisher Scientific), with VpxF1 and VprR1 primers containing either the P5 or P7 Illumina adaptors, each with a unique 8-nucleotide index sequence for multiplexing. The multiplexed samples were sequenced on a MiSeq instrument (Illumina) and analyzed as previously described.^36^^,^^37^

### RBC isolation

RBCs were isolated using Lymphoprep (StemCell) density gradient centrifugation at 800 × g for 15 min with the brake off. The RBC layer was transferred to a new tube and washed five times with RPMI to remove non-RBC populations. RBCs were cultured at 40% hematocrit in RPMI supplemented with 10% fetal bovine serum (FBS) (Cytiva), 1× penicillin-streptomycin (Gibco), and 1× L-glutamine (Gibco). RBC lysis experiments were performed by incubating 1 mL RBCs with 5 mL of hemolysis buffer (150 mM ammonium chloride [Thermo Fisher Scientific], 12 mM sodium bicarbonate [Sigma-Aldrich], 0.1 mM EDTA [Invitrogen]) for 30 min.

### Splenocyte cell culture

Cryopreserved splenocytes were thawed in 100 μg/mL DNAse I (Sigma) and resuspended in R10 media, composed of RPMI supplemented with 10% FBS (Cytiva), 1× penicillin-streptomycin (Gibco), and 1× L-glutamine (Gibco). If viability was <85%, dead cells were removed using the Dead Cell Removal kit (Miltenyi), according to the manufacturer’s protocols. T cells were isolated using the CD3 microbead positive-selection kit for NHP cells (Miltenyi), and monocytes were purified using the CD14 microbead positive-selection kit for NHP cells (Miltenyi), following manufacturer protocols. The flowthrough from T cell and monocyte isolations was processed using the EasySep non-human primate B cell isolation kit (StemCell) to isolate B cells. T cells were cultured at 2 × 10^6^ cells/mL in R10 supplemented with 50 U/mL IL-2 (PeproTech) every 2–3 days, with initial stimulation using NHP T cell Activation/Expansion Beads (Miltenyi) prepared per the manufacturer’s instructions. Monocytes were cultured at 1 × 10^6^ cells/mL in R10 supplemented with 25 ng/mL GM-CSF (Thermo Fisher Scientific) or 50 ng/mL M-CSF (R&D), which was replenished every 2–3 days. B cells were cultured at 2 × 10^6^ cells/mL in R10 supplemented with ImmunoCult-ACF Human B Cell Expansion Supplement (StemCell) every 2–3 days.

### Flow cytometry

Gene marking of PBMC and tissue cell subsets was assessed using the following fluorophore-conjugated antibodies: CD4 (clone OKT4, BioLegend), CD45 (clone D058-1283, BD), CD11b (clone ICRF44, BioLegend), P2RY12 (clone S16001E, BioLegend), CD8 (clone RPA-T8, BioLegend), CD3 (clone SP34-2, BD), CD20 (clone 2H7, BioLegend), CD56 (clone NCAM16.2, BD), CD14 (clone MϕP9, BD), and CD16 (clone 3G8, BD). For detection of eCD4Ig-Emm06 in T cells, cells were pre-incubated with 10 μg/mL Brefeldin A (Sigma-Aldrich) and 0.67 μL/mL GolgiStop (BD) for 4 h at 37°C. All other staining panels proceeded directly to live/dead staining (Invitrogen), followed by incubation with Fc Block (BD) for 10 min at room temperature and surface staining for 20 min at room temperature. Intracellular staining for eCD4Ig-Emm06 was performed by fixing cells with Cytofix/Cytoperm (BD) for 20 min at 4°C, followed by overnight staining at 4°C with antibodies against CD4 (clone L200, BD) and rhesus IgG2 (NHPRR). Before data acquisition, samples were washed and resuspended in PBS containing 2% FBS (Cytiva) and 1 mM EDTA (Gibco).

For RBC staining, cells were incubated with anti-rhesus RBC antibody (clone T3G6, StemCell) for 20 min at 4°C, followed by rat anti-mouse IgG1 (BD). Annexin V (eBioscience) staining was then performed for 15 min at room temperature, according to the manufacturer’s protocol. Cells were fixed with 0.05% glutaraldehyde (Sigma-Aldrich) for 10 min at 4°C and permeabilized with 0.1% Triton X-100 (Sigma-Aldrich) for 10 min at room temperature. Intracellular staining for eCD4Ig-Emm06 was performed as described above.

B cell and monocyte cell lines were stained for cell surface receptors by first staining with a live/dead stain (Invitrogen), followed by cell surface staining with the following fluorophore-conjugated antibodies for 30 min at 4°C: HLA-DR (clone G46-6, BD), CD16 (clone 3G8, BD), CD32 (clone AT10, Invitrogen), and CD64 (clone 10.1, Biolegend). All stains included fluorescence-minus-one controls. eCD4-Ig binding experiments included an incubation of cells with serial dilutions of recombinant eCD4-Ig, eCD4Ig-Emm06, or IgG2 isotype control (clone QA16A13, Biolegend) for 30 min at 4°C, before staining with Live/Dead dye (Invitrogen) and then anti-rhesus IgG2 (NHPRR) for 30 min at 4°C. For blocking experiments, cells were pre-incubated with or without anti-CD32 antibody (100 μg/mL, clone FLI8.26, BD) for 20 min at room temperature, followed by the direct addition of eCD4-Ig or eCD4Ig-Emm06 (100 μg/mL) with incubation for 30 min at 4°C, before proceeding to flow cytometry staining to measure binding. All B cell and monocyte cell line staining experiments were conducted in duplicate.

Flow cytometry data were collected on a BD LSRFortessa and analyzed using FlowJo software (BD).

### Serum cytokine measurements

NHP sera were measured for cytokines (T helper [Th] 1/Th2) using the NHP Th1/2 cytokine kit/cytometric bead array (CBA), following the manufacturer instructions (BD). Flow data were obtained using a BD LSR II flow cytometer, and results were quantified using the BD CBA FCAP software.

### ELISA

To quantify eCD4Ig-Emm06, 96-well high-binding U-bottom ELISA plates (Greiner) were coated overnight at 4°C with 8.3 μg/mL HIV BaL gp120 or SIVmac239 gp130 (NIH HIV Reagent Program, Division of AIDS, NIAID; HRP-20082, contributed by DAIDS, NIAID; ARP-12797, contributed by Dr. Klaus Uberla) in ELISA Carbonate Coating Buffer (Thermo Fisher Scientific). Plates were washed three times with PBS-T (PBS +0.05% Tween 20) and blocked for 1 h at 37°C with a 1:1 mixture of StartingBlock Blocking Buffer (Thermo Fisher Scientific) and 10% BSA in PBS. Standard curves were generated by serially diluting recombinant eCD4Ig-Emm06 in blocking buffer and adding to the plates in duplicate. Serum samples were diluted 1:200 in blocking buffer and added in duplicate. Supernatant samples were measured at dilutions ranging from 1:1 to 1:3. Plates were incubated for 2 h at 37°C, then washed ten times with PBS-T. Horseradish peroxidase (HRP)-conjugated anti-monkey IgG antibody (Sigma-Aldrich) was added at a 1:5,000 dilution and incubated for 1 h at room temperature, followed by six washes with PBS-T. Plates were developed by incubation with 1-Step TMB Substrate (Thermo Fisher Scientific) for 15 min, and the reactions were stopped with 0.02 N sulfuric acid (VWR). Absorbance at 450 nm was measured using a BioTek EL800 plate reader, and analyses were performed using GraphPad Prism 10.

Anti-eCD4Ig-Emm06 antibodies were quantified using an ELISA modified to coat ELISA plates with 5 μg/mL eCD4Ig-Emm06, and secondary antibodies were changed to a combination of goat anti-human kappa-HRP and goat anti-human lambda-HRP (Southern Biotech), each at a 1:5,000 dilution. Standard curves were generated using serial dilutions of ibalizumab (Thermo Fisher Scientific). Serum samples were diluted 1:100 in blocking buffer and measured in duplicate.

Serum anti-gp41 antibody titers were measured using the ELISA described above, with several modifications. ELISA plates were coated with SIVmac239 gp41 (ImmunoDX). Serum samples were initially diluted 1:100, followed by serial 2-fold dilutions. Endpoint titers were defined as the highest dilution with an optical density 450 (OD_450_) greater than the pre-SIV challenge OD_450_.

Anti-HDAd IgG and IgM titers were measured using the published ELISA format, modified to coat plates with HDAd 6/3+ virus (5 × 10^8^ viral particles per well).^6^ The secondary antibodies were either HRP-conjugated anti-monkey IgG or IgM antibodies (Sigma-Aldrich) diluted 1:5,000. Titers were defined as the antibody dilution at which the OD_450_ signal was reduced by 50% (IC50 titers).

### SIVmac239 neutralization

SIVmac239 virus for *in vitro* assays was produced from 293T cells. 293T cells were cultured in DMEM supplemented with 10% cosmic calf serum (Cytiva), 1× penicillin-streptomycin (Gibco), 1× L-glutamine (Gibco), 1× sodium pyruvate (Gibco), and 1× non-essential amino acids (Gibco). Cells were seeded at 12 × 10^6^ cells per 150 mm plate and transfected 24 h later with the SIVmac239 SpX plasmid (NIH HIV Reagent Program, Division of AIDS, NIAID, ARP-12247, contributed by Dr. Ronald C. Desrosiers) using PEI Max (Polysciences) at a 3:1 PEI:DNA (w/w) ratio. Transfection proceeded for 12 h, after which the transfection media was replaced with fresh medium. Virus-containing supernatants were harvested 48 h later, with removal of cellular debris by centrifugation at 1,500 × g for 10 min, followed by filtration through 0.22 μm flasks (Millipore). Virus aliquots were stored at −80°C.

For neutralization assays, NHP serum was heat-inactivated at 56°C for 1 h and serially diluted (3-fold) in DMEM supplemented with 10% cosmic calf serum (Cytiva), 1× penicillin-streptomycin (Gibco), and 1× L-glutamine (Gibco). Serum dilutions were incubated with SIVmac239 at 37°C for 1 h, using a virus concentration titrated to produce a relative luminescence units (RLU) at least 10-fold above background, in accordance with the Los Alamos National database neutralization assay recommendations. In parallel, TZM-bl cells were seeded at 10,000 cells per well in the presence of 8.4 μg/mL DEAE-dextran in 96-well, tissue culture-treated, clear-bottom plates. The cells were mixed with the serum-virus mixture in duplicate following the 1 h incubation. After 48 h, luciferase activity was measured using the luciferase assay system (Promega), following the manufacturer’s instructions. Luminescence was detected using a BioTek Synergy H4 Hybrid Reader (Agilent).

### Statistics

Data are presented as mean ± SEM. The Student’s *t* test was used for comparisons between two groups. For comparisons of multiple groups, one- or two-way ANOVA tests were performed with corrections for multiple comparisons. Statistical analyses were performed using GraphPad Prism version 10.0.3 (GraphPad Software, Inc.). *p* values less than 0.05 were considered significant.

## Data and code availability

All original data are available from the authors without any restrictions. SIVmac239 Env sequences are available on Gene Expression Omnibus (GEO) (series GSE305698). The software script for analyzing SIVmac239 Env sequences is accessible on Github (https://github.com/jbui2-ui/SIV-Env-Sequence-Analysis).

## Acknowledgments

The study was supported by 10.13039/100000002NIH grants R01 AI174304 (A.L.), U19HL156247 (H.-P.K.), P51OD010425 (H.-P.K.), K08AI183990 (J.K.B.), and a grant from the 10.13039/100000865Bill and Melinda Gates Foundation (BMGF), INV-017692 (A.L.). Under the grant conditions of the BMGF, a Creative Commons Attribution 4.0 Generic License has already been assigned to the Author Accepted Manuscript version that may arise from this submission.

This project has been funded in part with federal funds from the 10.13039/100000054National Cancer Institute, National Institutes of Health, under contract no. 75N91019D00024. The content of this publication does not necessarily reflect the views or policies of the Department of Health and Human Services, nor does mention of trade names, commercial products, or organizations imply endorsement by the U.S. Government.

This research was supported by the Flow Cytometry Shared Resource, RRID:SCR_022613, of the 10.13039/100005895Fred Hutch/10.13039/100007812University of Washington/Seattle Children’s Cancer Consortium (P30 CA015704).

The WaNPRC is supported by the 10.13039/100000002National Institutes of Health (NIH) Office of Research Infrastructure Programs (ORIP) under award no. P51OD010425 and U42OD011123. Plasma SIV viral loads and subset assays were run by the WaNPRC Virology & Immunology Core (V&IC), led by Sandra Dross and supported by the above WaNPRC awards and the Cell Analysis Facility Flow Cytometry Shared Resource Lab in the Department of Immunology at the University of Washington.

Mass Spectrometry analyses were performed by Young Ah Goo, Byoung-Kyu Cho, and Jong Hee Song at the Mass Spectrometry Technology Access Center at the McDonnell Genome Institute (MTAC@MGI) at Washington University School of Medicine, supported by the 10.13039/100007878Diabetes Research Center/10.13039/100000002NIH grant P30 DK020579, 10.13039/100007930Institute of Clinical and Translational Sciences/NCATS CTSA award UL1 TR002345, and 10.13039/100014571Siteman Cancer Center/NCI CCSG grant P30 CA091842.

We thank Jeffrey D Lifson for insightful discussions on SIVmac239, Melissa Berg and Alexandria Christodoulou for veterinarian supervision, Robert Murnane for pathology analyses, and Laurence Stensland for performing tissue viral load quantification.

## Author contributions

A.L., H.-P.K., and J.K.B. provided the conceptual framework for the study. A.L., C.L., A.-S.K., M.F., K.R.J., B.F.K., H.-P.K., and J.K.B. designed the experiments. C.L., A.K.A., V.N., A.G., H.W., A.G., S.G., J.M.-R., A.R., S.S.R., J.K., D.P., N.S., C.B., C.M.F., A.L., and J.K.B. performed the experiments. M.F. and M.D.A. provided the eCD4Ig-Emm06 construct. C.M.F. and B.F.K. provided the SIVmac239M2 virus and performed barcode sequencing. N.S., C.B., T.O., and M.F. performed Env sequencing. D.P. and P.N. generated HDAd vectors for NHP infusion. C.L., A.-S.K., P.N., M.D.A., K.R.J., and H.-P.K. provided critical comments on the manuscript. J.K.B. and A.L. wrote the manuscript.

## Declaration of interests

A.L. and H.-P.K. are academic co-founders of Ensoma Bio. H.-P.K serves as Scientific Advisor for Ensoma Bio. M.D.A., C.B., and M.F. have significant financial interests in Emmune, Inc. Emmune, Inc. owns and has exclusive licenses to patents and patent applications pertaining to eCD4-Ig.

## Supplemental information

Document S1. Figures S1–S22 and Table S1

Document S2. Article plus supplemental information
