# Supplementary material for: In vivo HSC gene therapy enables sustained eCD4-Ig expression for SIV prevention
Source: Mol Ther Adv. 2026 Feb 26;34(1):201683. doi: 10.1016/j.omta.2026.201683 (PMC13148906; doi:10.1016/j.omta.2026.201683)
Supplement: Document S1. Figures S1–S22 and Table S1 [file mmc1.pdf]

OMTA, Volume *34*

Supplemental information

In vivo HSC gene therapy enables sustained eCD4-Ig expression for SIV prevention

Chang Li, Anna K. Anderson, Anne-Sophie Kuhlmann, Veronica Nelson, Audrey Germond, Hongjie Wang, Aphrodite Georgakopoulou, Sucheol Gil, Jasmin Martinez-Reyes, Andrew Riker, Shruthi Shankar Raman, Jiho Kim, Philip Ng, Donna Palmer, Michael D. Alpert, Nickolas Skamangas, Charles Bailey, Tianling Ou, Christine M. Fennessey, Michael Farzan, Keith R. Jerome, Brandon F. Keele, Hans-Peter Kiem, André Lieber, and John K. Bui

Table S1. NHP Cohort Summary. Two groups of three rhesus macaques each received HDAd-control or HDAd-eCD4Ig-Emm06. After at least 12 weeks elapsed from the final O^6BG/BCNU selection, sequential SIV challenges were initiated to assess protective efficacy.

Animal ID	Sex	Weight (kg)	Age	Interval between O^6BG/BCNU and SIV challenge
**HDAd-control**				
A21137	M	4.1	3y10m	12.7wk
A23051	M	5.4	4y0m	12.1wk
A22065	M	6.3	4y9m	23wk
**HDAd-eCD4Ig-Emm06**				
A21138	F	2.5	2y8m	12.1wk
A21140	M	2.1	3y2m	12.1wk
A22054	M	7.2	5y4m	12.9wk

Table S2. Genotypes of SIV controller alleles. MHC genotypes were determined by sequencing exon 2 amplicons. Animals carrying alleles associated with SIV control, indicated by an asterisk (*), were balanced between groups.

Animal ID	MHC-A Haplotype 1	MHC-A Haplotype 2	MHC-B Haplotype 1	MHC-B Haplotype 2
**HDAd-control**				
A21137	A004.01	A006.01	B001.01	B024.01
A23051	A002.01*	A004.01	B017.01*	B069.01
A22065	A004.01	A028.01	B002.01	B017.01
**HDAd-eCD4Ig-Emm06**				
A21138	A004.01	A012.01	B001.01	B001.01
A21140	A002.01*	A023.01	B012.01	B028.01
A22054	A019.01	A123.01	B043.01	B043.03

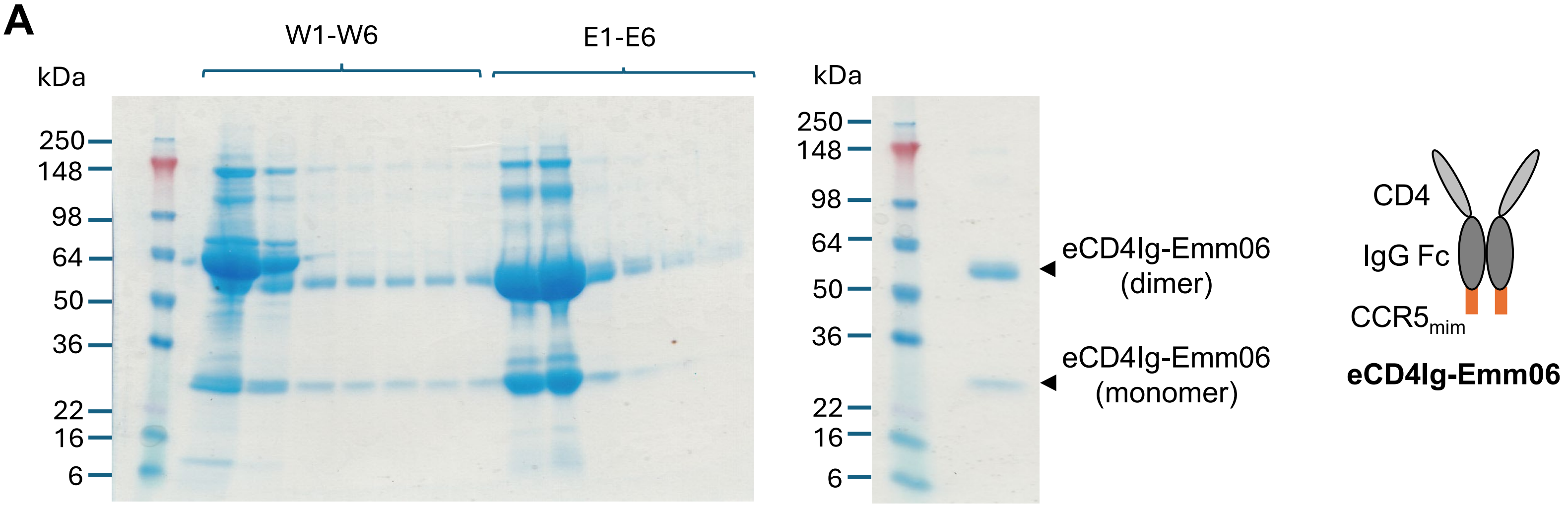

B

	Mass (Da)	Intensity	DScore
○	96,444	22.64	40.81
▽	96,524	64.52	65.67
△	96,599	20.69	7.76
▷	96,645	34.37	36.92
□	96,735	20.70	35.87
◇	96,814	49.97	53.42
☆	96,892	25.76	16.96
○	96,943	32.85	29.25
▽	96,978	31.83	28.7
△	97,024	43.18	51.66
▷	97,105	100.00	68.18
□	97,145	54.33	17.72
◇	97,184	51.98	20.25
☆	97,225	59.67	43.37
○	97,403	19.00	4.23
▽	97,473	17.53	24.9

Fig.S1 *In vitro* characterization of recombinant eCD4Ig-Emm06. **A)** SDS-PAGE gel of purification fractions of eCD4Ig-Emm06 protein. Supernatants were collected from 293T cells four days after transduction with HDAd-eCD4Ig-Emm06**.** Supernatant was mixed 1:1 with wash buffer (0.15M NaCl, 20mM Na_2HPO_4, pH 7.0) before addition of Protein A resin slurry. Six wash fractions of 1 column volume (CV) (W1-W6) were collected before eluting protein with six fractions of 1 CV elution buffer (E1-E6) (0.1M glycine, pH 3.0). Elutions 1-5 were pooled and dialyzed in phosphate-buffered saline (PBS, pH 7.4) and concentrated before running final product on the second gel (right panel). All fractions and proteins were run under reducing conditions and boiled at 98˚C prior to loading on 4-20% Tris-Glycine gels. The panel also shows a schematic of the eCD4Ig-Emm006 protein (non-reduced dimer). **B)** Intact protein mass spectrometry analysis of eCD4-Ig sulfation corresponding to Figure 1F, including intensity and D-score values.

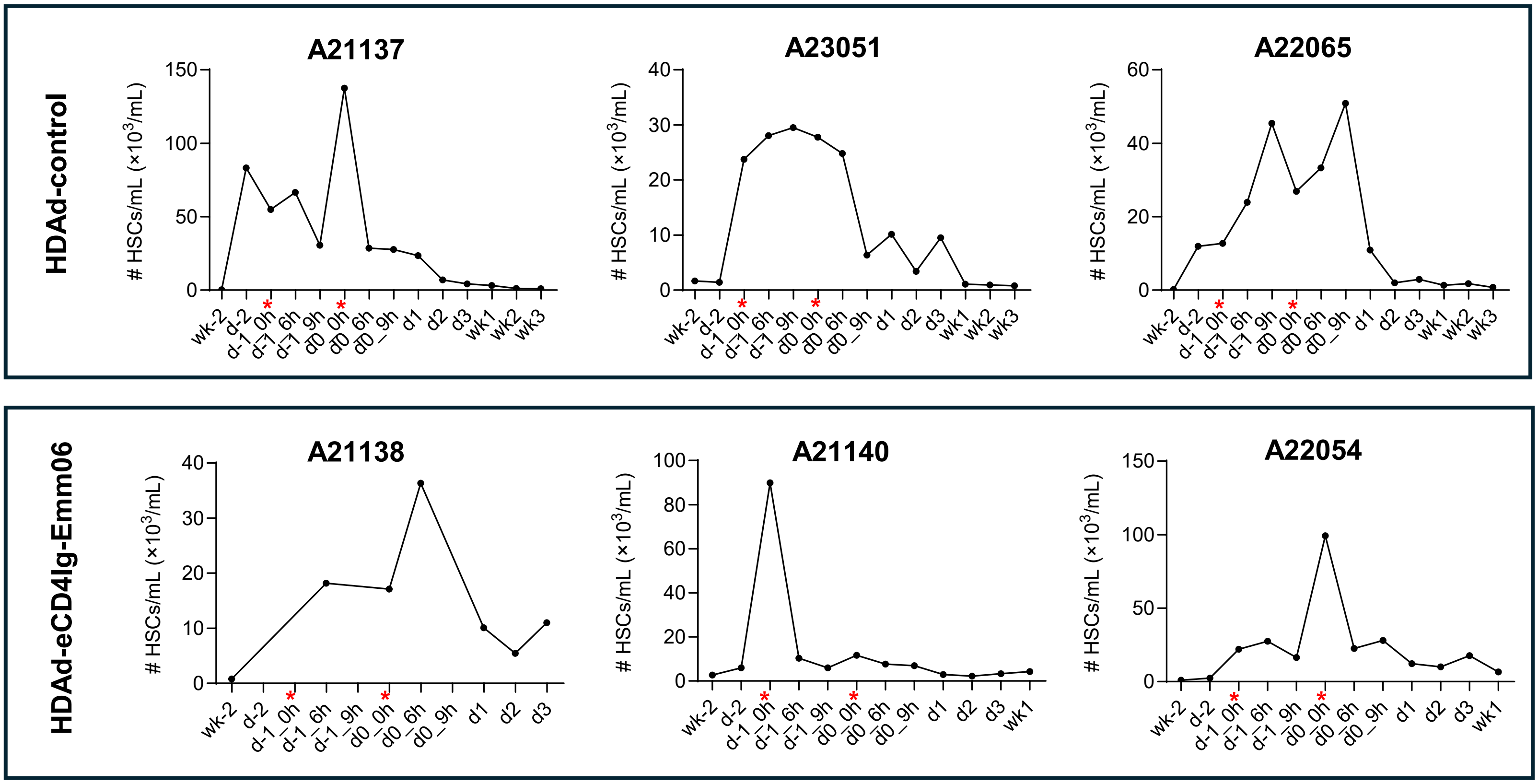

Fig.S2 Mobilization efficacy in rhesus macaques. Animals were mobilized with G-CSF and AMD3100, as previously described.[7] The number of mobilized CD34+/CD45RA-/CD90+ cells ("HSCs") per ml blood was measured by flow cytometry. HDAd was injected 8 hours after AMD3100 indicated by a red star (★) at day -1, 0 hours and day 0, 0 hours.

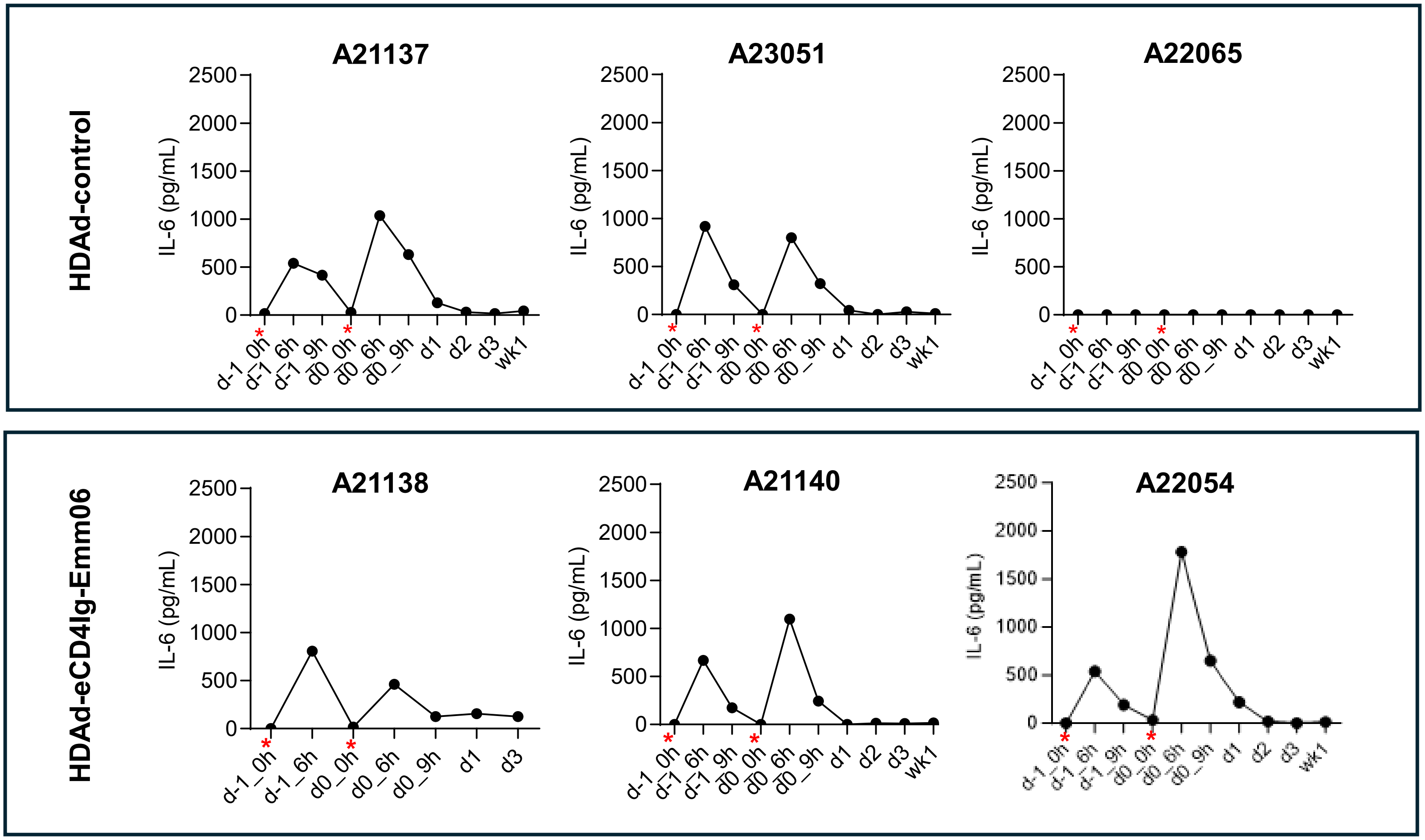

Fig.S3 Transient mild serum IL-6 elevations following each HDAd infusion. Serum cytokines were measured by cytometric bead array. HDAd was injected at day -1, 0 hours and day 0, 0 hours (labeled by a red star). IL-6 was detectable in 5 of 6 animals with transient elevations following each HDAd infusion, whereas other cytokines (IL-2, IL-4, IL-5, TNF-α, IFN-γ) remained below the detection limit (20 pg/mL).

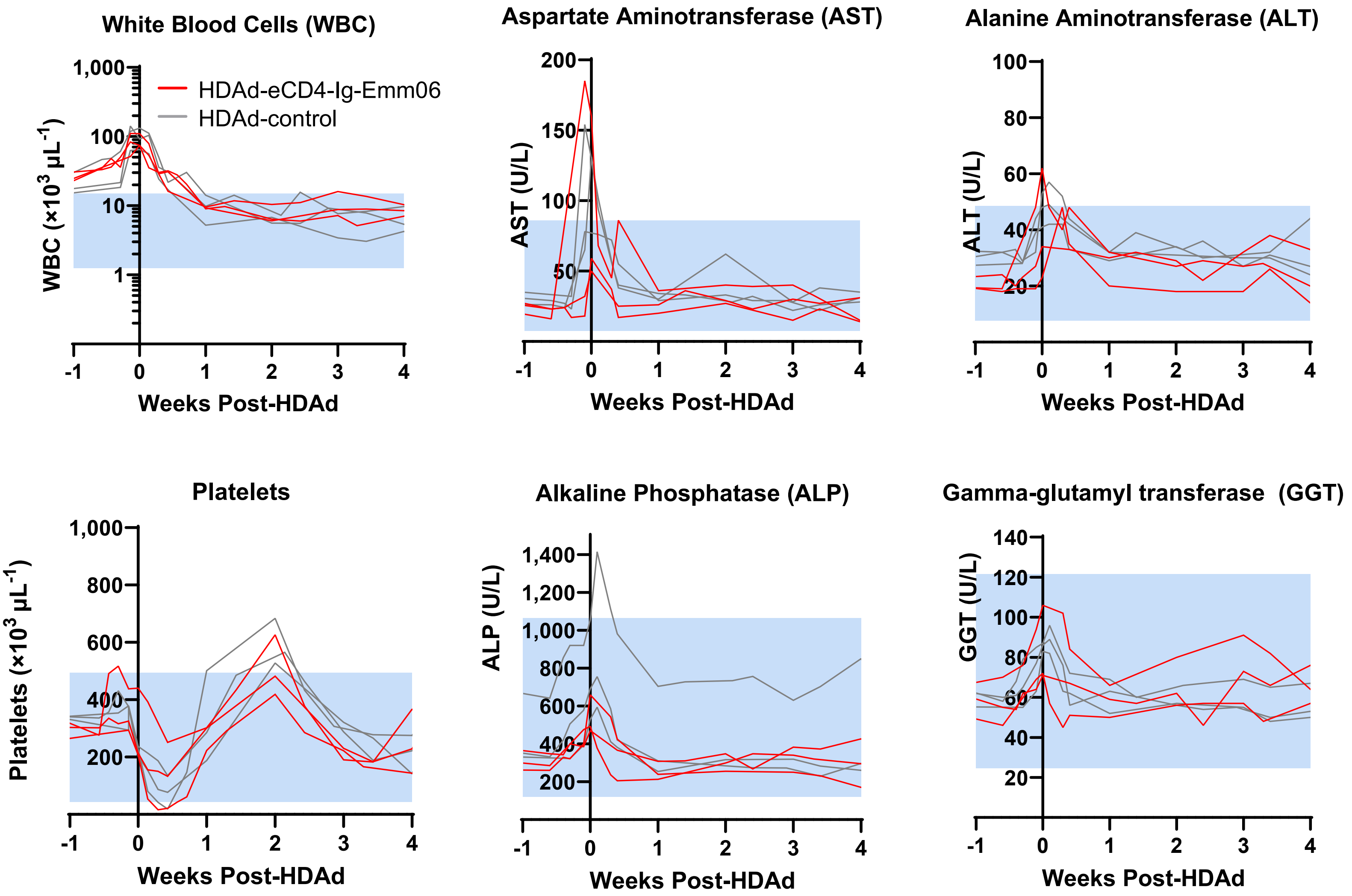

Fig.S4 Normalization of platelet counts, white blood cell counts, and hepatic function tests within 1 week of HDAd infusion. HDAd was injected at day -1, 0 hours and day 0, 0 hours. Reference intervals are shown in blue shading.

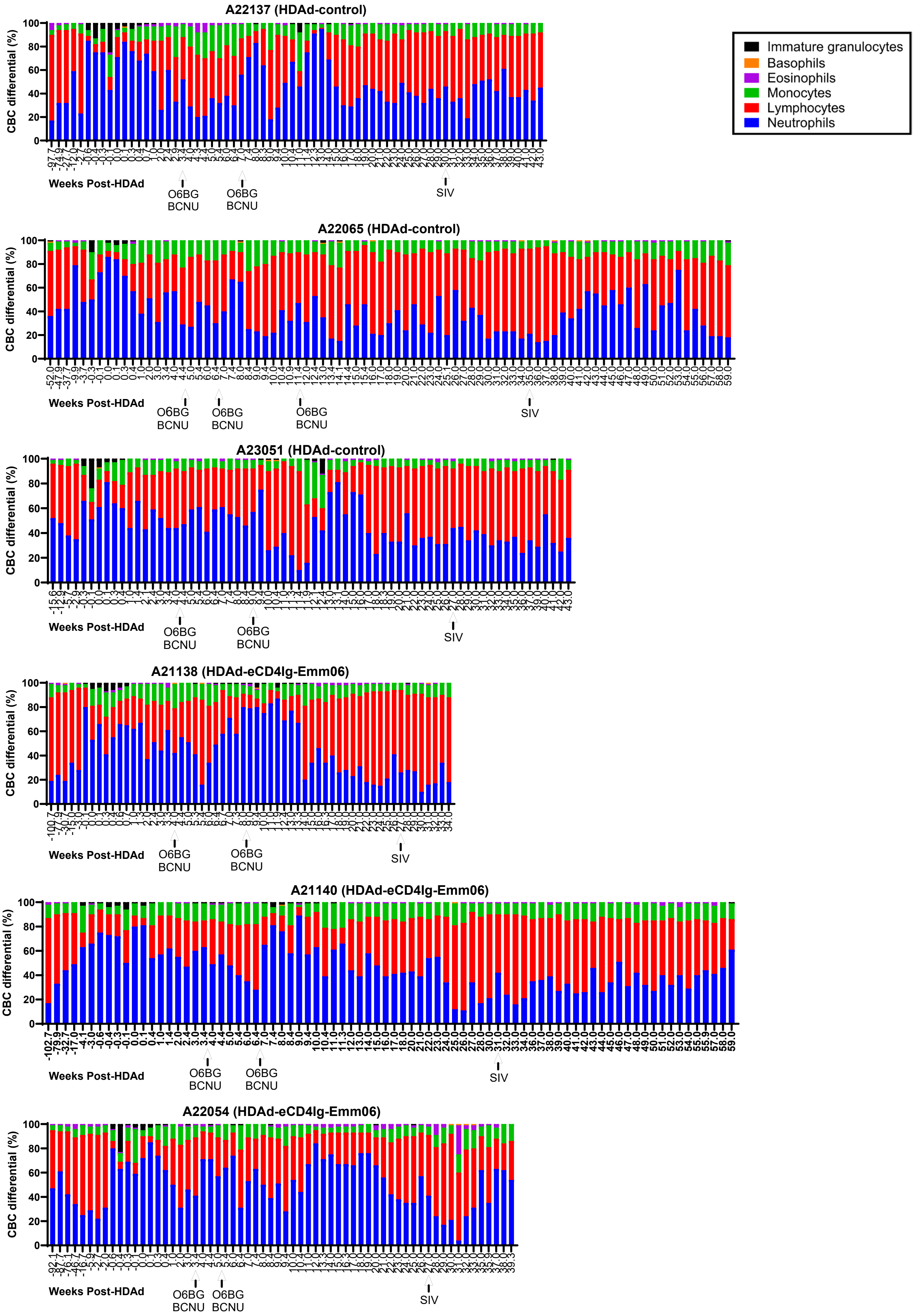

Fig.S5. Differential blood cell counts for HDAd-control and HDAd-eCD4Ig-Emm06 injected animals. O^6BG/BCNU treatment and SIV challenge are indicated by arrows. Leukocyte subsets were measured by complete blood count differentials. No significant depletion of leukocyte subsets was observed following HDAd or O^6BG/BCNU administration.

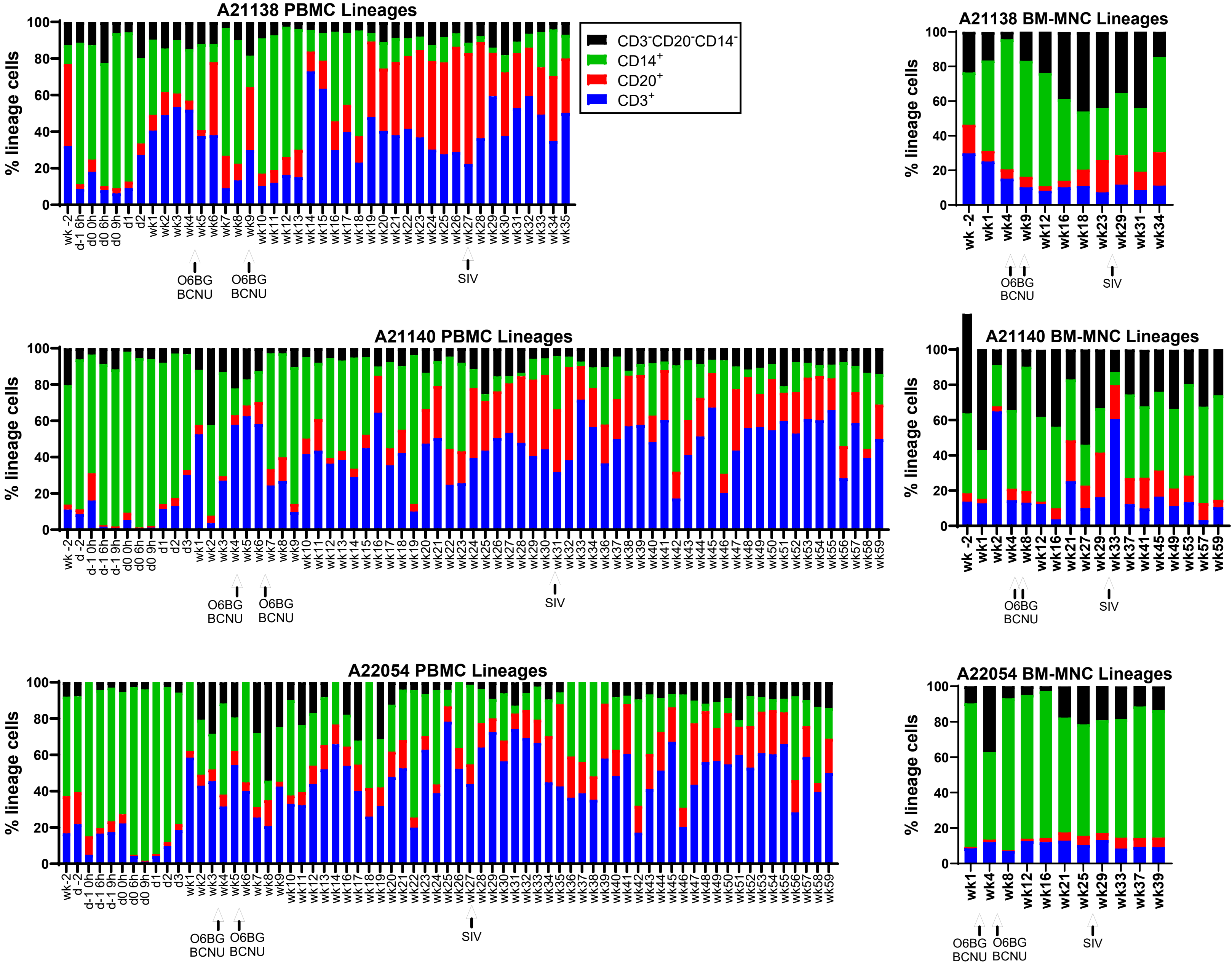

Fig.S6. Percentage of lineage-positive cells in HDAd-eCD4Ig-Emm6 injected animals. Shown are data for $CD3^+$ (T-cells), $CD20^+$ (B-cells), and $CD14^+$ (monocytes) as well as $CD3^-$/$CD20^-$/$CD14^-$ cells in PBMCs (left panels) and bone marrow mononuclear cells (right panels), as measured by flow cytometry. O^6BG/BCNU treatment and SIV challenge are indicated by arrows. No significant cytopenias were observed after HDAd or O^6BG/BCNU administration.

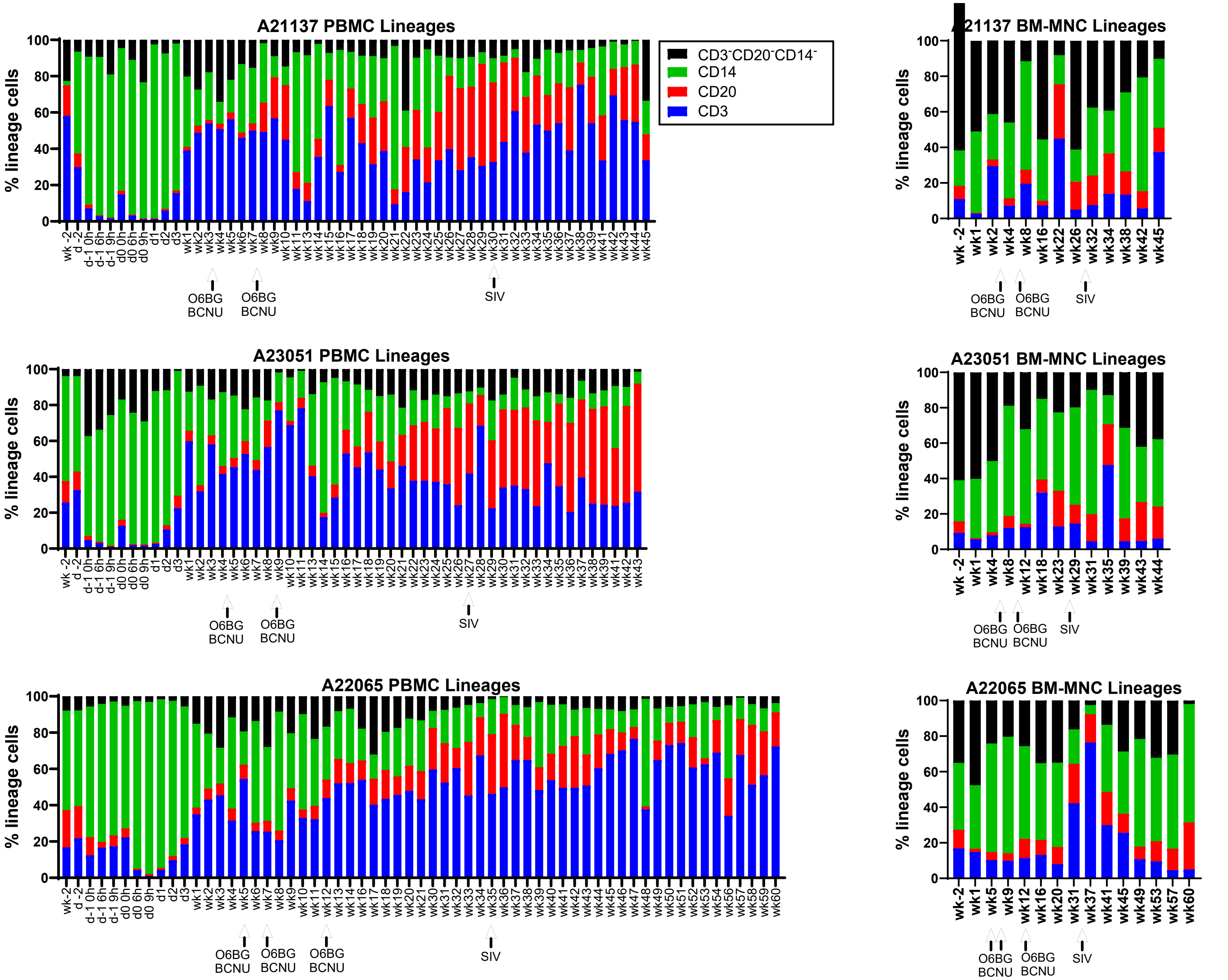

Fig.S7. Percentage of lineage-positive cells in HDAd-control injected animals. Shown are data for $CD3^+$ (T-cells), $CD20^+$ (B-cells), and $CD14^+$ (monocytes) as well as $CD3^-/CD20^-/CD14^-$ cells in PBMCs (left panels) and bone marrow mononuclear cells (right panels), as measured by flow cytometry. O^6BG/BCNU treatment and SIV challenge are indicated by arrows. No significant cytopenias were observed after HDAd or O^6BG/BCNU administration.

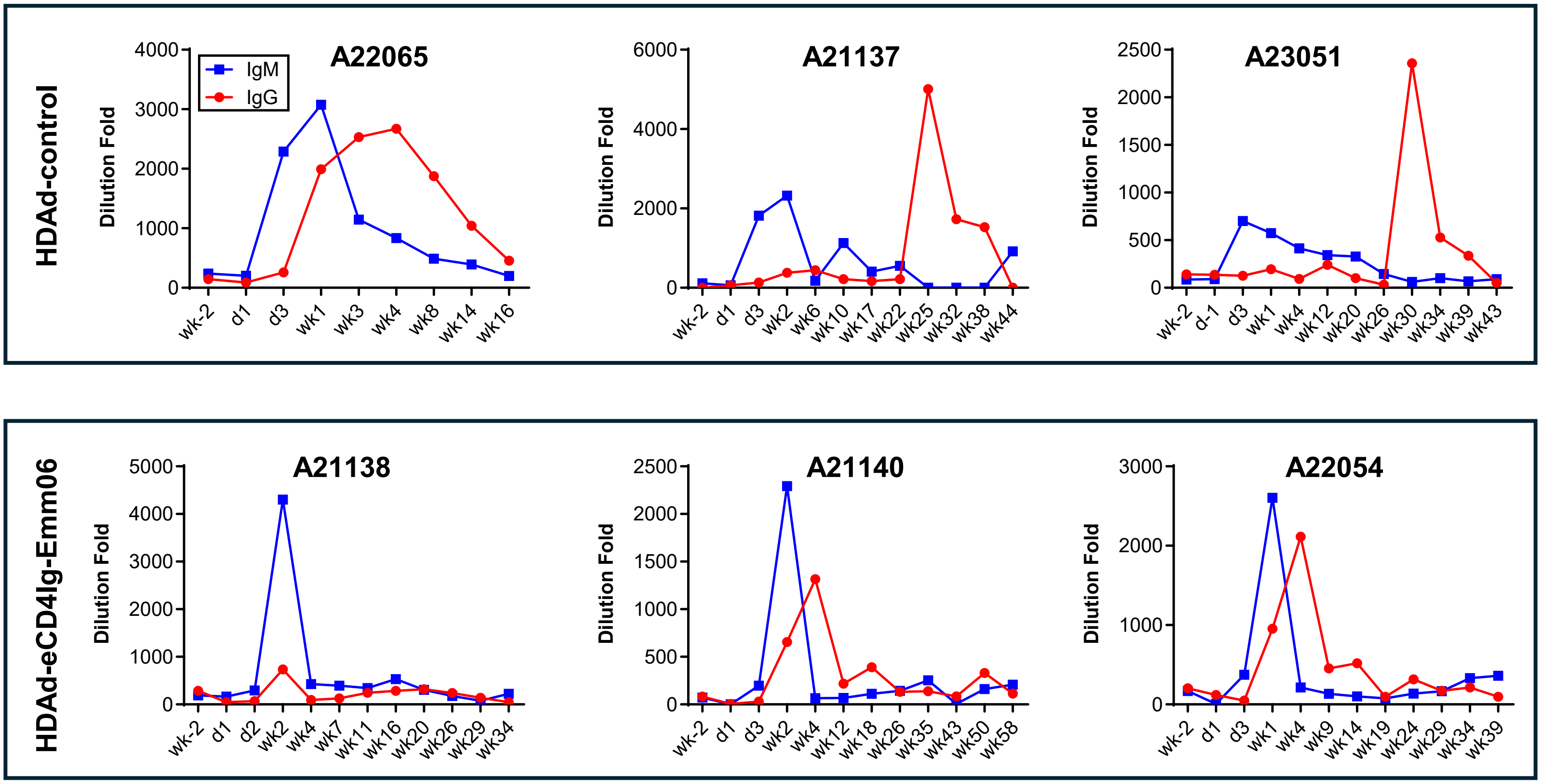

Fig.S8. Titers of serum IgM and IgG antibodies against HDAd6/3+ viral particles. wk-2 are pretreatment titers. HDAd was injected on day -1 and day 0. IgM (blue) and IgG (red) titers are the antibody dilution by which the OD450 signal was reduced by 50% (half maximal inhibitory concentration [IC50] titers).

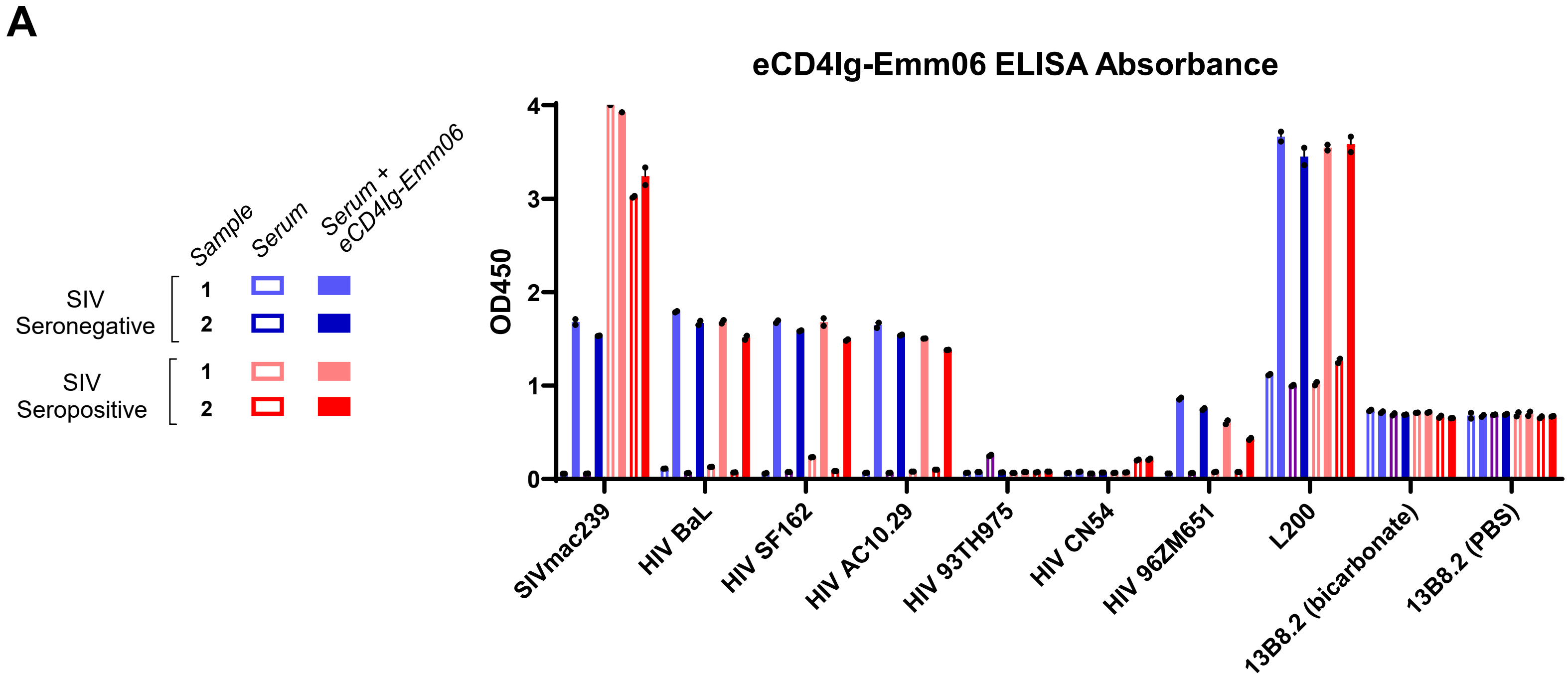

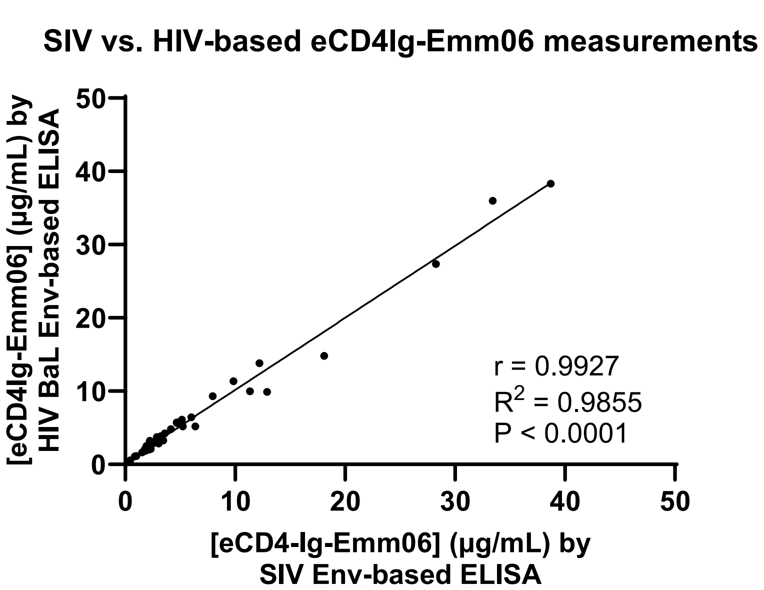

Fig.S9. eCD4Ig-Emm06 ELISA design and validation. A) ELISA plates were coated with various capture reagents to detect eCD4-Ig-Emm06. Serum from NHPs (n=2) were collected before and after SIV seroconversion and spiked with recombinant eCD4-Ig-Emm06. OD450 was measured in duplicate to identify assay designs that specifically detect eCD4Ig-Emm06 without cross-reactivity to anti-SIV antibodies. Error bars represent S.E.M. **B)** SIV and HIV-based ELISA measurements of serum eCD4Ig-Emm06 levels in NHPs treated with HDAd-eCD4Ig-Emm06 were significantly correlated by Pearson correlation ($p < 0.0001$).

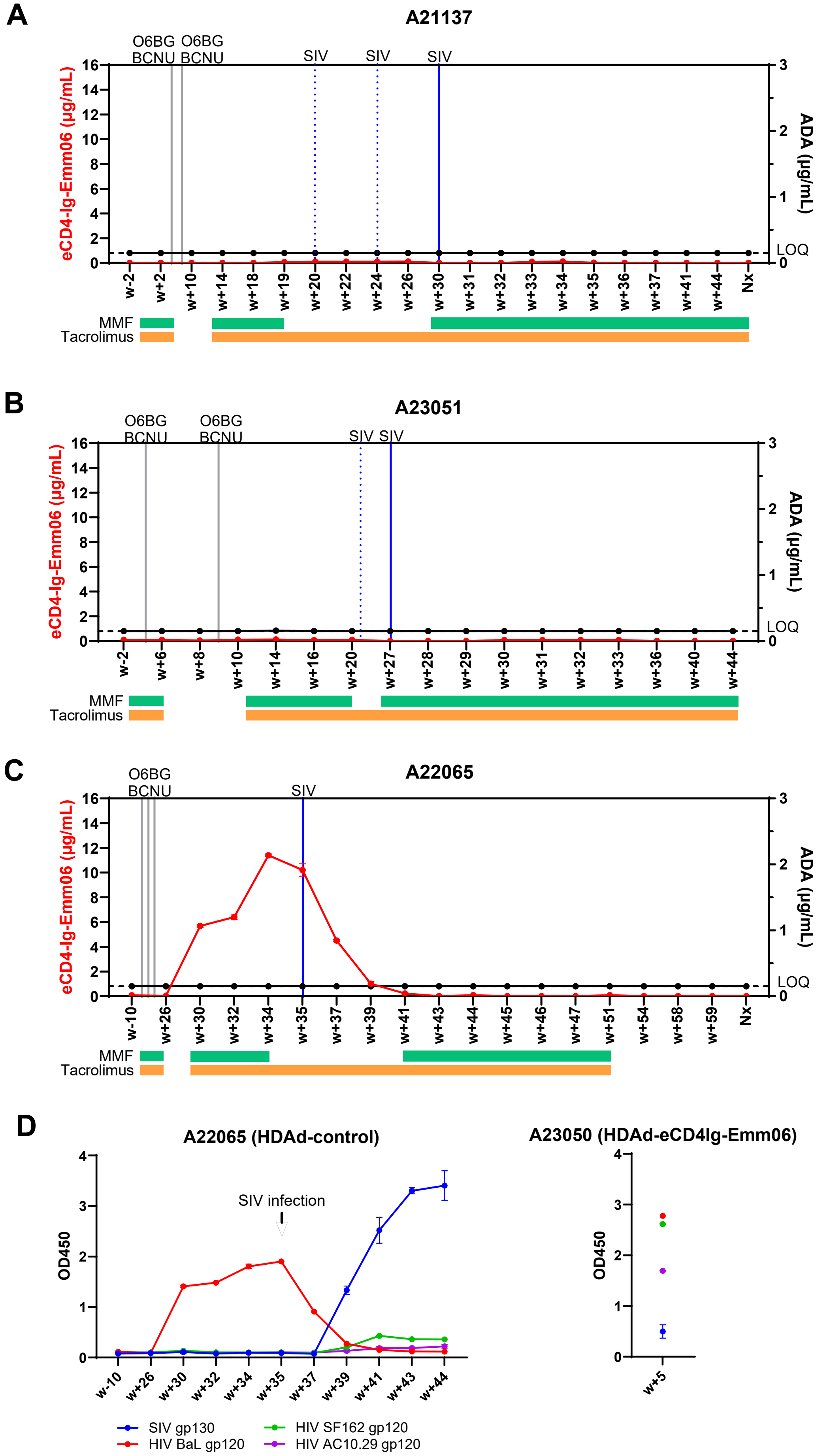

Fig.S10. Serum eCD4Ig-Emm06 concentrations and ADA levels in animals injected with HDAd-control. No serum eCD4Ig-Emm06 or ADA were detected in A211137 **(A)** and A23051 **(B)**. In A22065 **(C)**, eCD4Ig-Emm06 was detectable between weeks 26 and 39 without ADA. **(D)** The eCD4Ig-Emm06 signal in A22065 was determined to be a transient false-positive, given the absence of absorbance in ELISAs coated with SIV and HIV Env proteins that were validated for eCD4Ig-Emm06 detection, as shown in Fig. S9A. As a positive control, OD450 was also measured using serum from an uninfected animal treated with HDAd-eCD4Ig-Emm06.

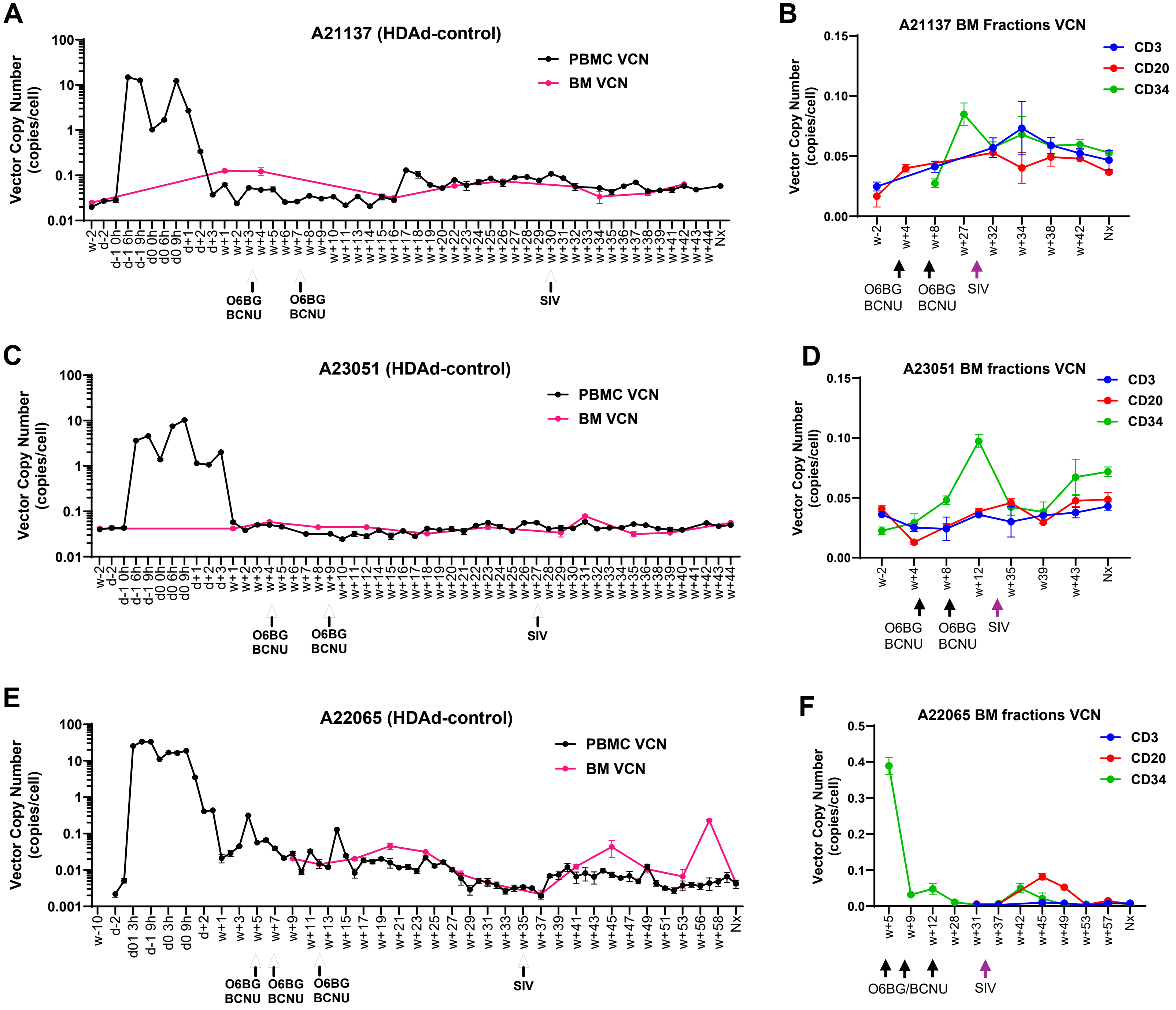

Fig.S11. Vector copy numbers (VCN) in HDAd-control injected animals. A, C, E) VCN per cell in PBMCs and BM MNCs. **B, D, F)** VCN per cell in bone marrow CD3⁺, CD20⁺ and CD34⁺ cell fractions. VCN are shown as mean values of triplicate measurements with S.E.M. error bars.

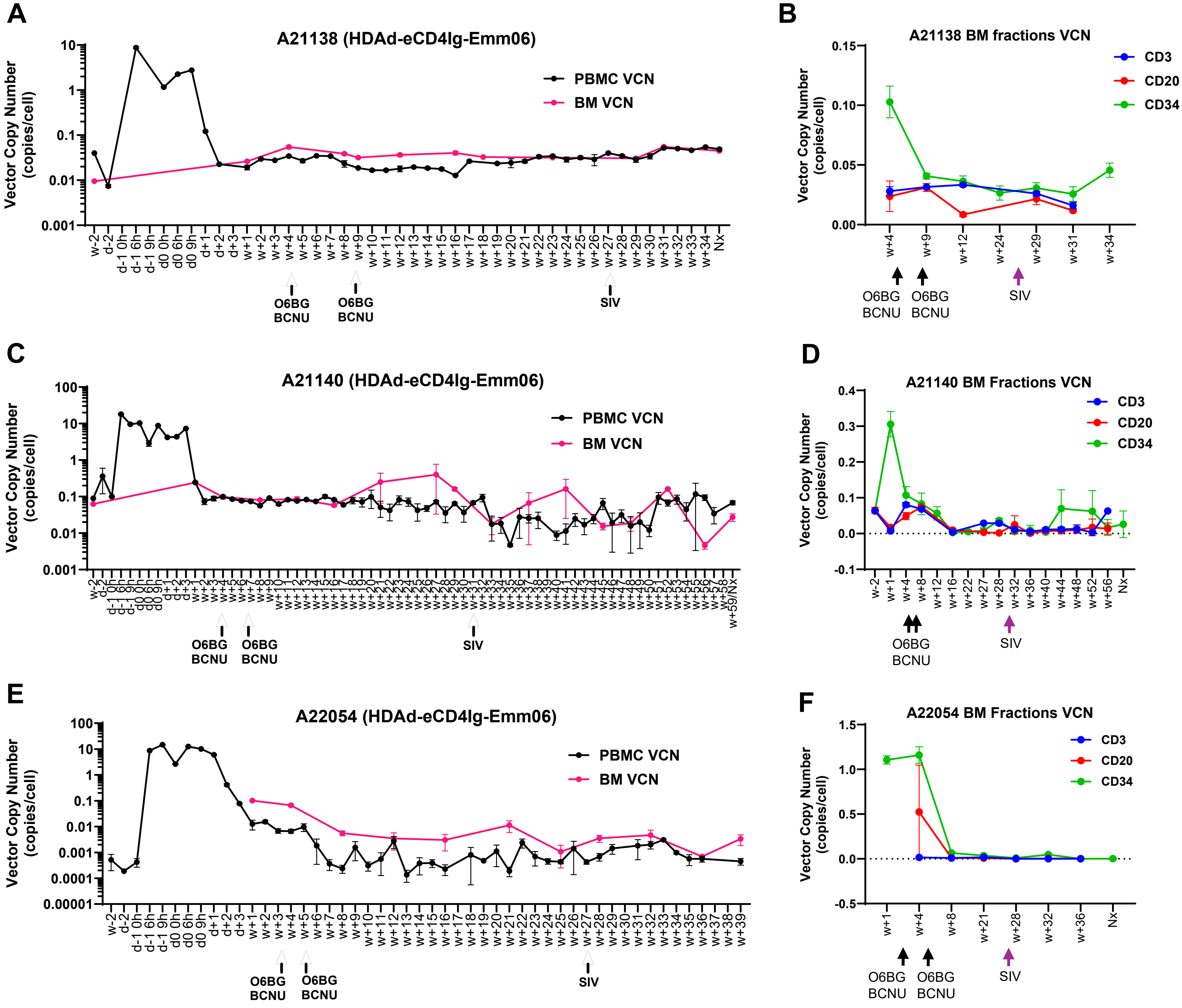

Fig.S12. Vector copy numbers (VCN) in HDAd-eCD4Ig-Emm06 injected animals. A, C, E) VCN per cell in PBMCs and BM MNCs. **B, D, F)** VCN per cell in bone marrow $CD3^{+}$, $CD20^{+}$ and $CD34^{+}$ cell fractions. VCN are shown as mean values of triplicate measurements with S.E.M. error bars.

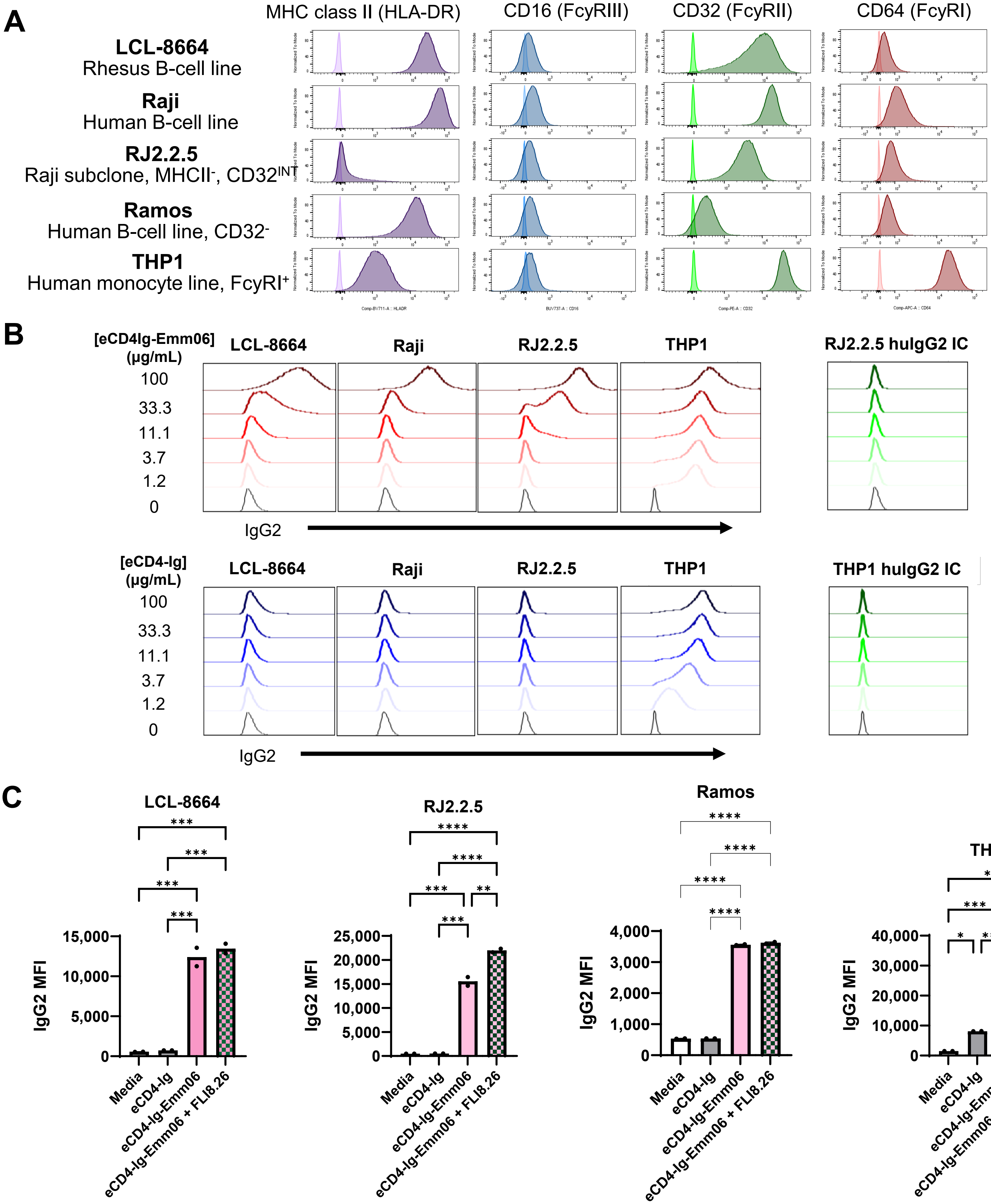

Fig.S13. eCD4Ig-Emm06 exhibits increased non-specific binding. A) Rhesus (LCL-8664) and human B-cell lines (Raji, RJ2.25 [MHCII-, CD32INT], and Ramos [CD32-]) and a human monocyte line (THP1) were assessed for expression of potential off-target receptors for eCD4Ig-Emm06 by flow cytometry. Staining was performed in duplicate and shown are representative histograms overlaid with fluorescence-minus-one (FMO) controls. **B)** Cell lines were incubated in duplicate with varying concentrations of recombinant eCD4-Ig or eCD4Ig-Emm06. A rhesus IgG2 isotype control was unavailable so a human IgG2 isotype control (huIgG2 IC) was used instead. eCD4-Ig and eCD4Ig-Emm06 binding was measured by staining for the IgG2 domain of eCD4-Ig by flow cytometry, with representative histograms shown. **C)** Cell lines were incubated in duplicate with recombinant eCD4-Ig, eCD4-Ig-Emm06, or eCD4-Ig-Emm06 with anti-CD32 block (FLI8.26 clone), each at 100 μg/mL. Binding is indicated by IgG2 MFI measured by flow cytometry, with error bars representing S.E.M. Statistical comparisons were performed using the one-way ANOVA with the Tukey correction for multiple comparisons, with only significant differences shown: * $p < 0.05$, ** $p < 0.01$, *** $p < 0.001$, **** $p < 0.0001$.

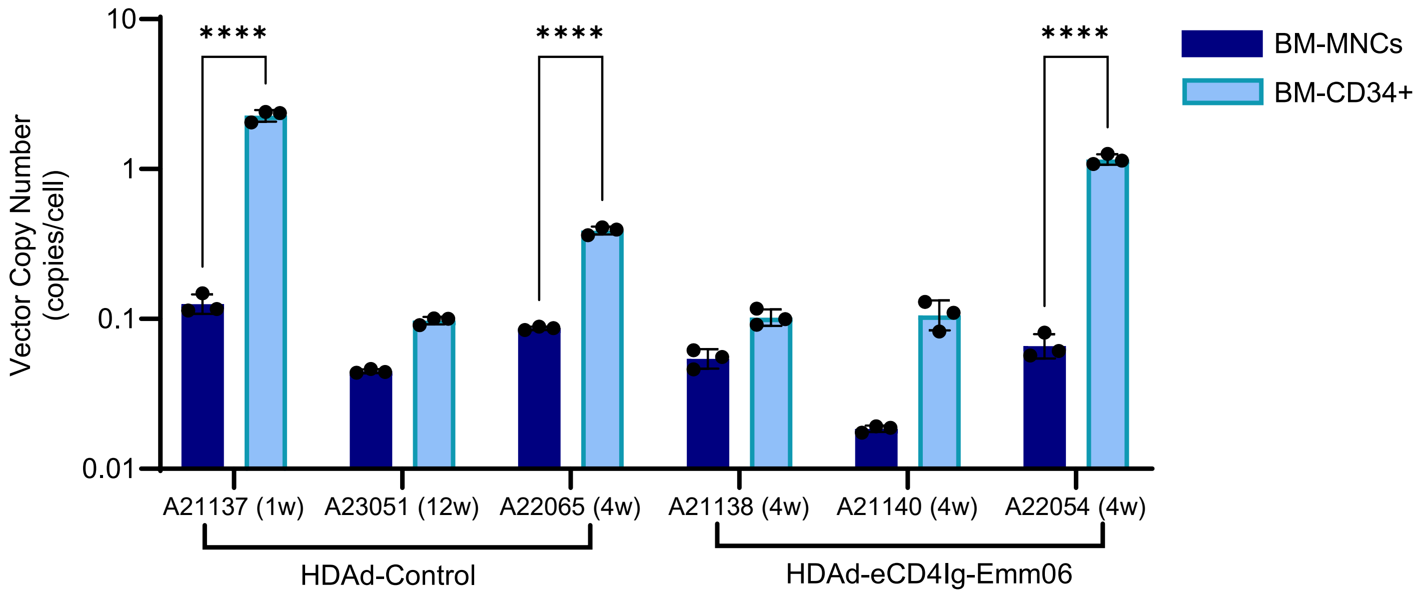

Fig.S14. Preferential Transduction of CD34+ cells in Bone Marrow. VCN was measured in triplicate from bone marrow mononuclear cells (BM-NC) and CD34+ cells (BM-CD34+) at the indicated time points following infusion of HDAd-eCD4Ig-Emm06 or HDAd-control. Statistical comparisons were performed using the two-way ANOVA with Šídák's multiple comparisons correction (* $p < 0.05$, ** $p < 0.01$, *** $p < 0.001$, **** $p < 0.0001$.).

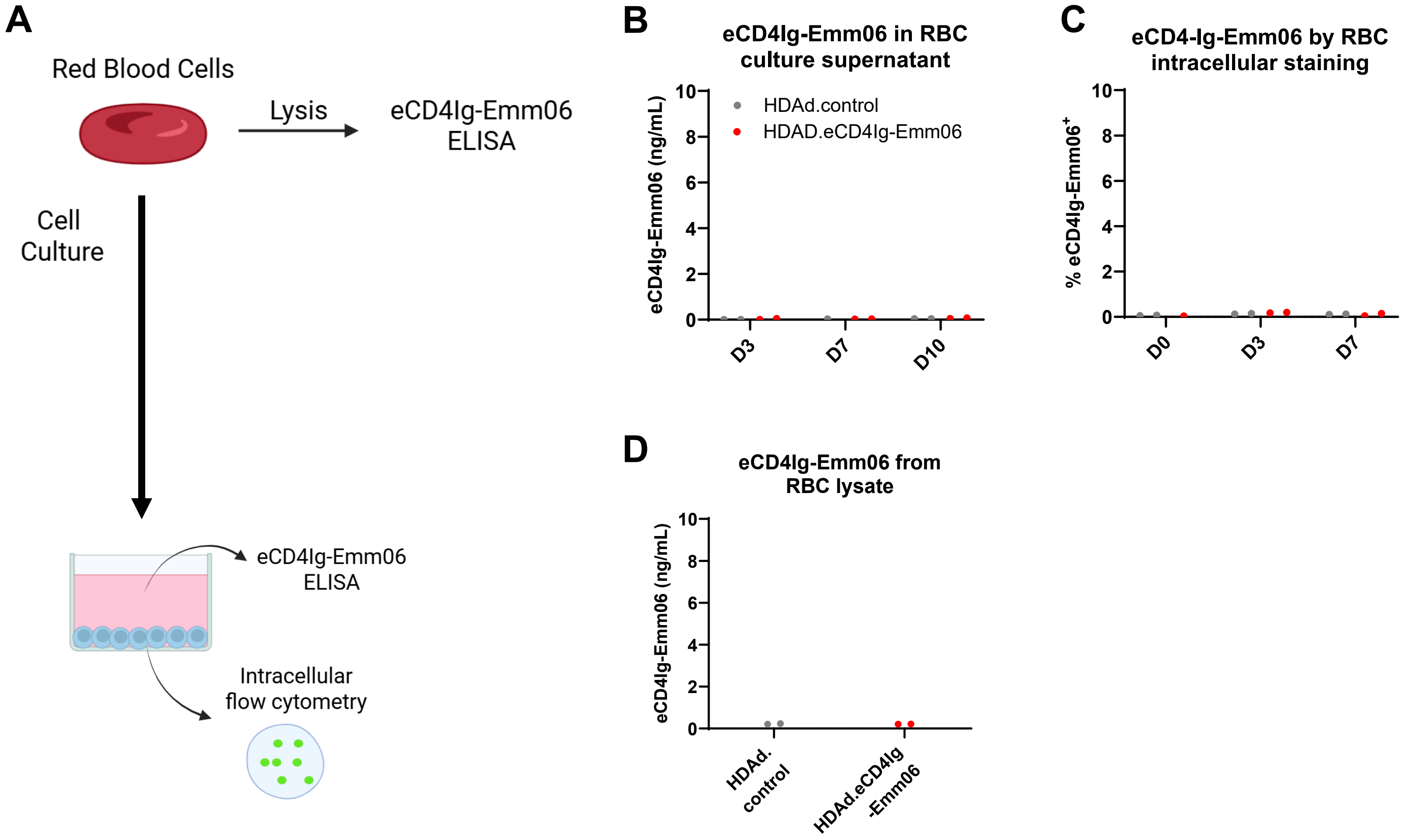

Fig.S15. eCD4Ig-Emm06 expression from peripheral RBCs. A) Schematic of RBC assays to measure eCD4Ig-Emm06 expression, created in https://BioRender.com. RBCs were isolated from NHPs that received HDAd-eCD4Ig-Emm06 or HDAd-control (n=2 each). **A, B)** RBCs were lysed and eCD4Ig-Emm06 release was measured by ELISA. **A, C, D)** RBCs were cultured *ex vivo* to evaluate for eCD4Ig-Emm06 production that was released into supernatant as measured by ELISA **(C)**, or expressed intracellularly as measured by flow cytometry **(D)**.

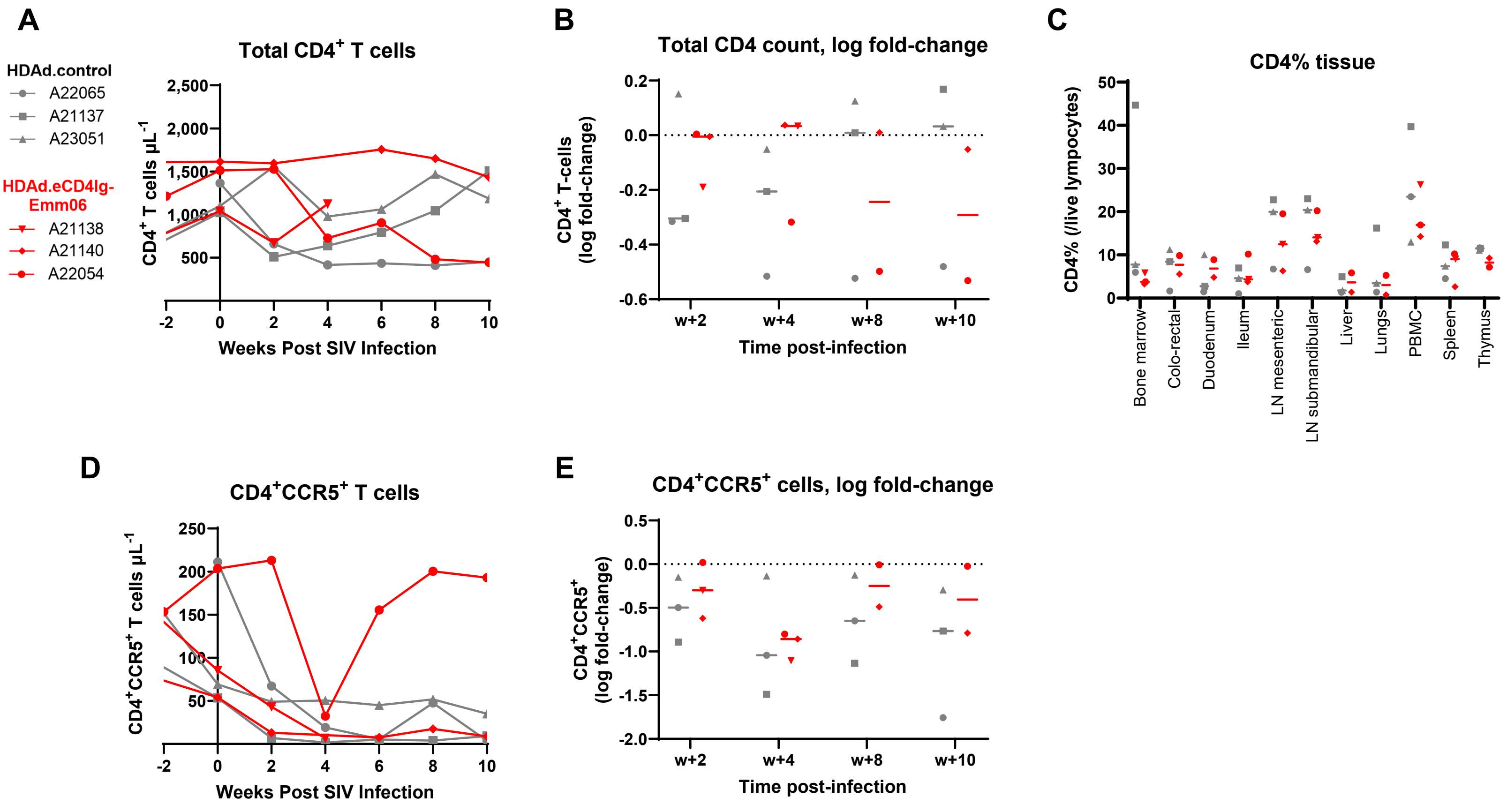

Fig.S16. CD4 counts following SIV infection. Rhesus macaques received HDAd-eCD4Ig-Emm06 (n=3, red) or HDAd-control (n=3, grey) and were intravenously inoculated with escalating doses of SIVmac239 until infection. **A, B)** $CD4^+$ T-cell counts are shown as absolute cell counts **(A)** and log fold-change relative to pre-infection **(B)**. **C)** The frequency of $CD4^+$ T-cells was measured in necropsy tissues. **D, E)** $CD4^+CCR5^+$ T-cell counts are shown as absolute cell counts **(D)** and log fold-change relative to pre-infection **(E)**. No significant differences were observed between groups in Fig. S16B, S16C, and S16E by two-way ANOVA with Šídák's multiple comparisons correction.

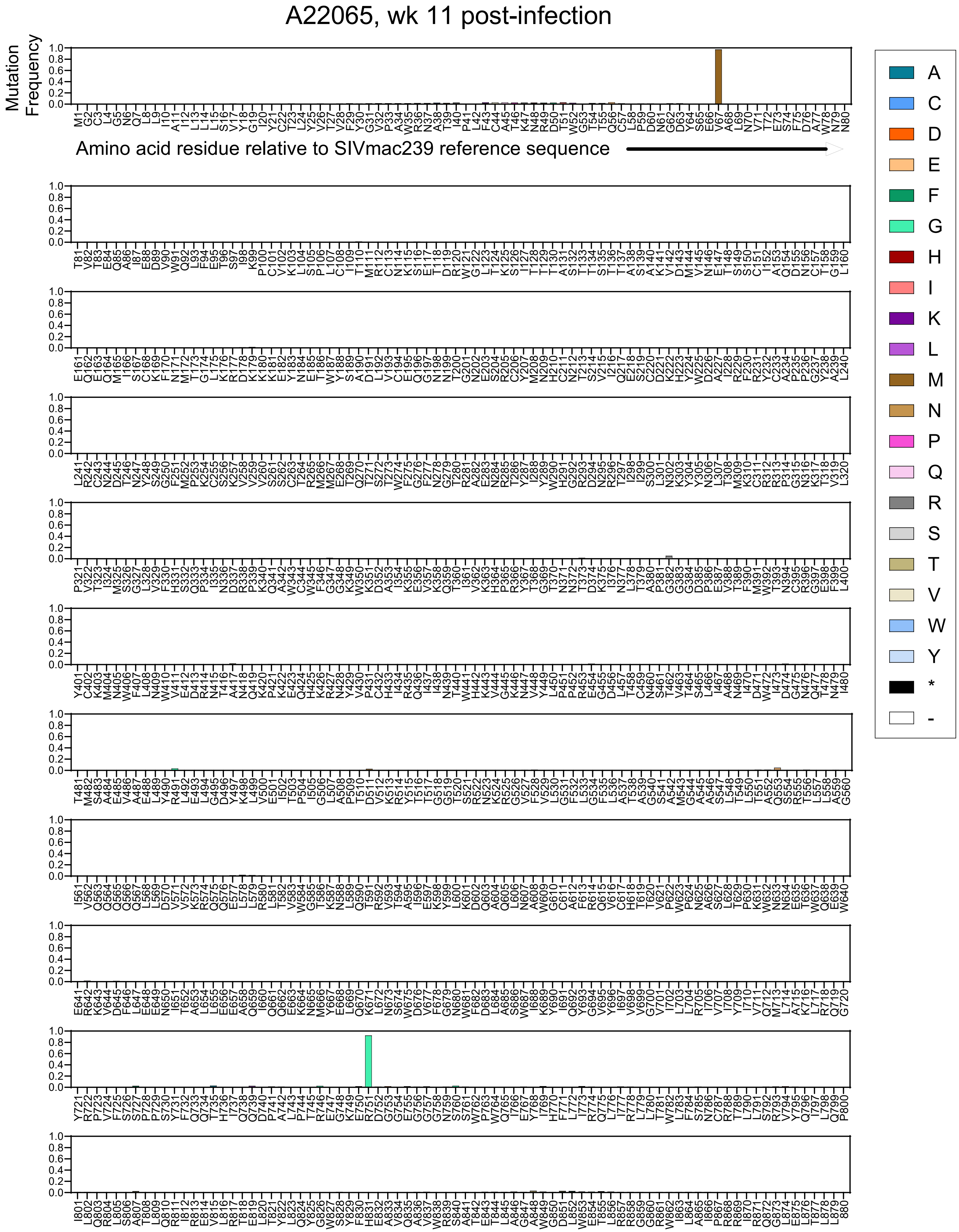

Fig.S17. A22065 Plasma SIV Env sequences. Plasma SIVmac239 viruses from 11 weeks post-infection were sequenced for the Env gene by long-read sequencing. Open reading frames were aligned to the SIVmac239 reference sequence (GenBank: M33262) by an in-house deep sequencing analysis pipeline. Amino acid mutation frequencies are plotted, with each mutation indicated by a color corresponding to the legend insert.

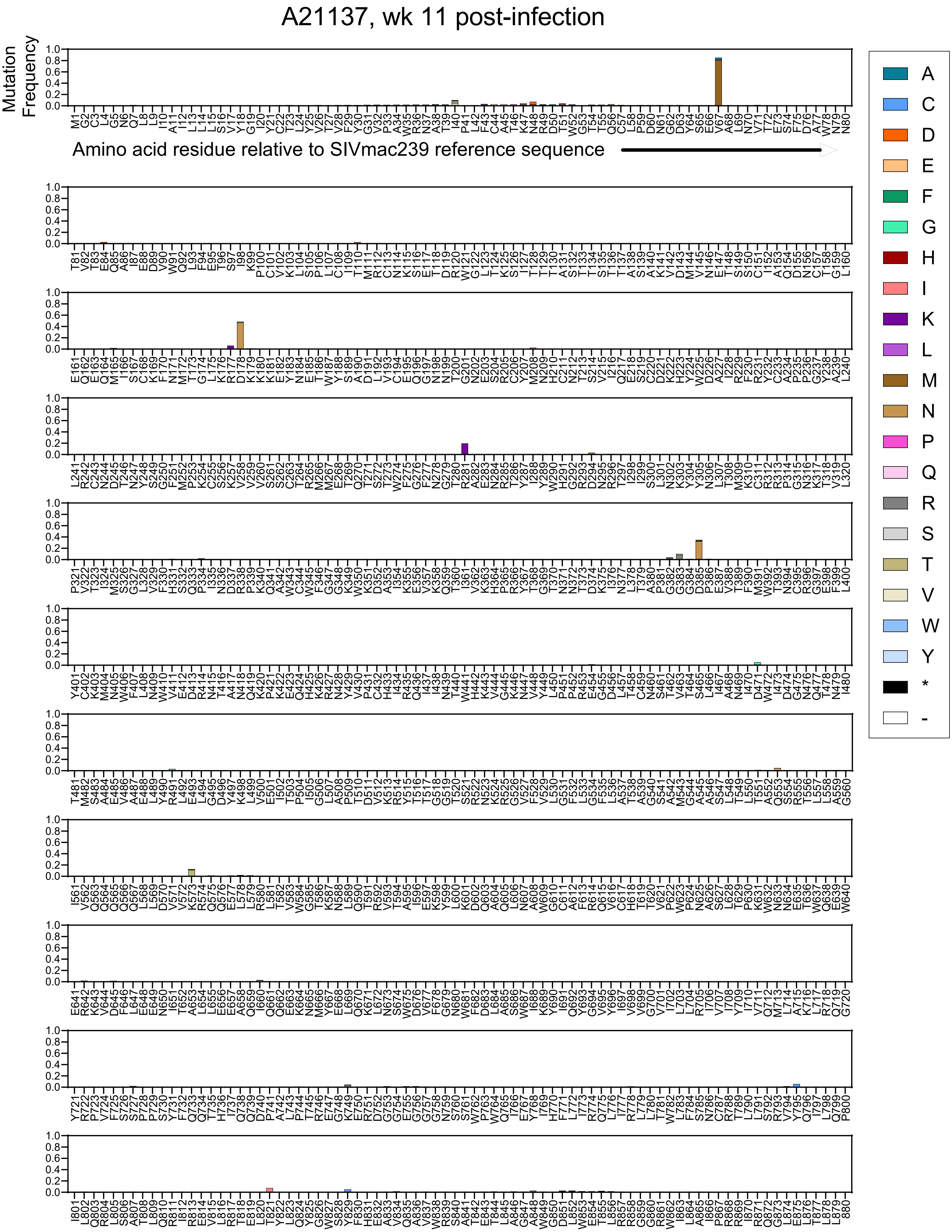

Fig.S18. A21137 Plasma SIV Env sequences. Plasma SIVmac239 viruses from 11 weeks post-infection were sequenced for the Env gene by long-read sequencing. Open reading frames were aligned to the SIVmac239 reference sequence (GenBank: M33262) by an in-house deep sequencing analysis pipeline. Amino acid mutation frequencies are plotted, with each mutation indicated by a color corresponding to the legend insert.

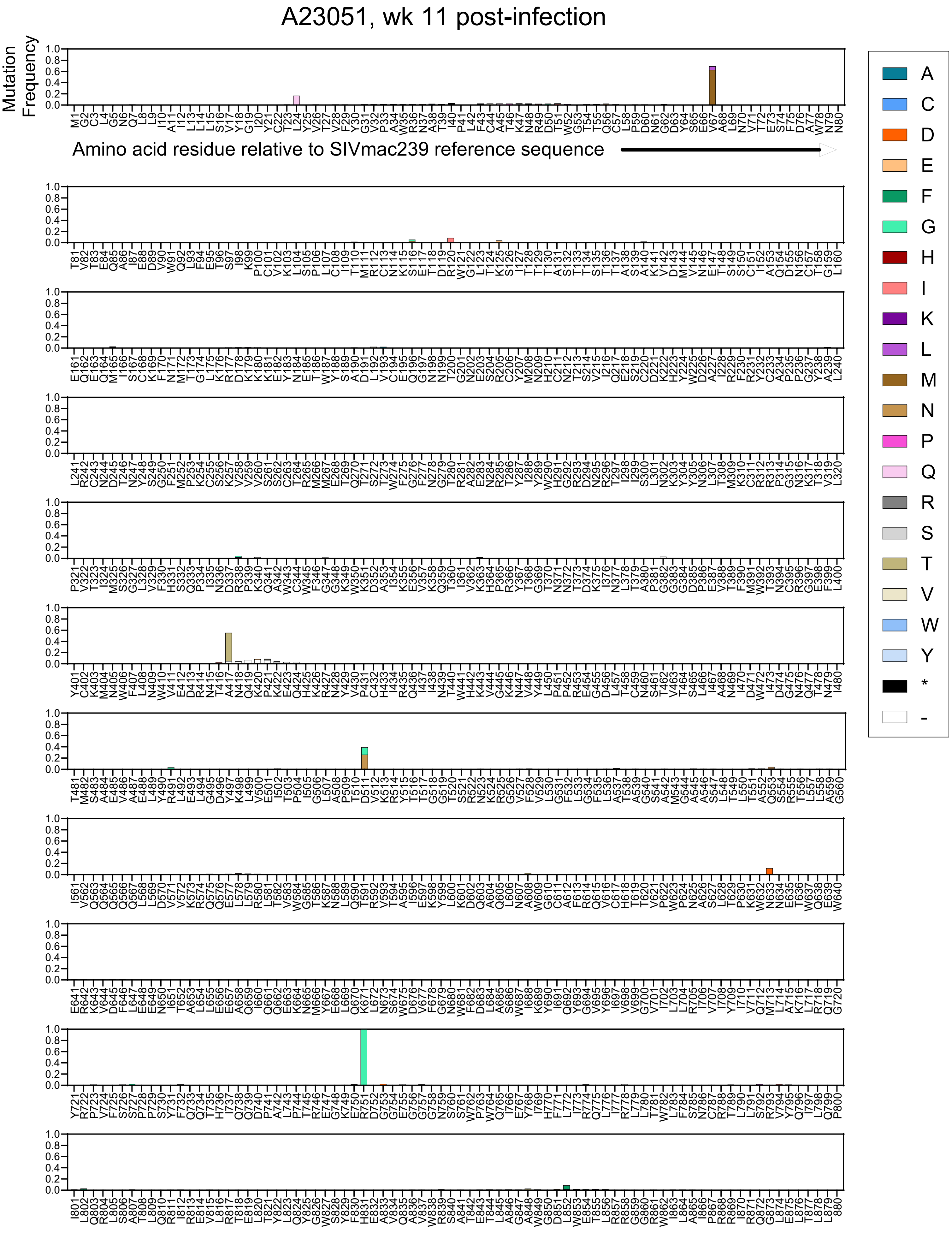

Fig.S19. A23051 Plasma SIV Env sequences. Plasma SIVmac239 viruses from 11 weeks post-infection were sequenced for the Env gene by long-read sequencing. Open reading frames were aligned to the SIVmac239 reference sequence (GenBank: M33262) by an in-house deep sequencing analysis pipeline. Amino acid mutation frequencies are plotted, with each mutation indicated by a color corresponding to the legend insert.

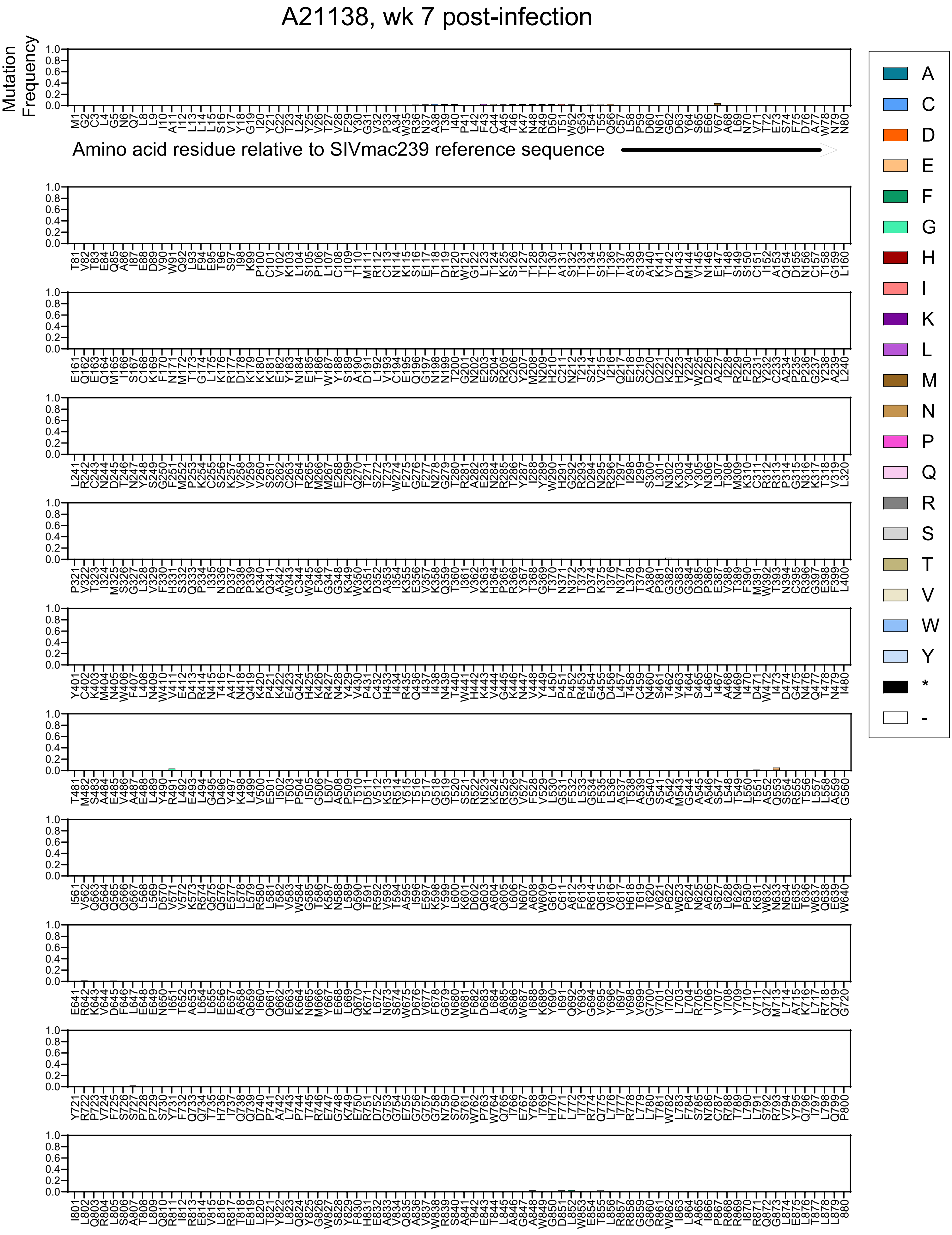

Fig.S20. A21138 Plasma SIV Env sequences. Plasma SIVmac239 viruses from 7 weeks post-infection were sequenced for the Env gene by long-read sequencing. Open reading frames were aligned to the SIVmac239 reference sequence (GenBank: M33262) by an in-house deep sequencing analysis pipeline. Amino acid mutation frequencies are plotted, with each mutation indicated by a color corresponding to the legend insert.

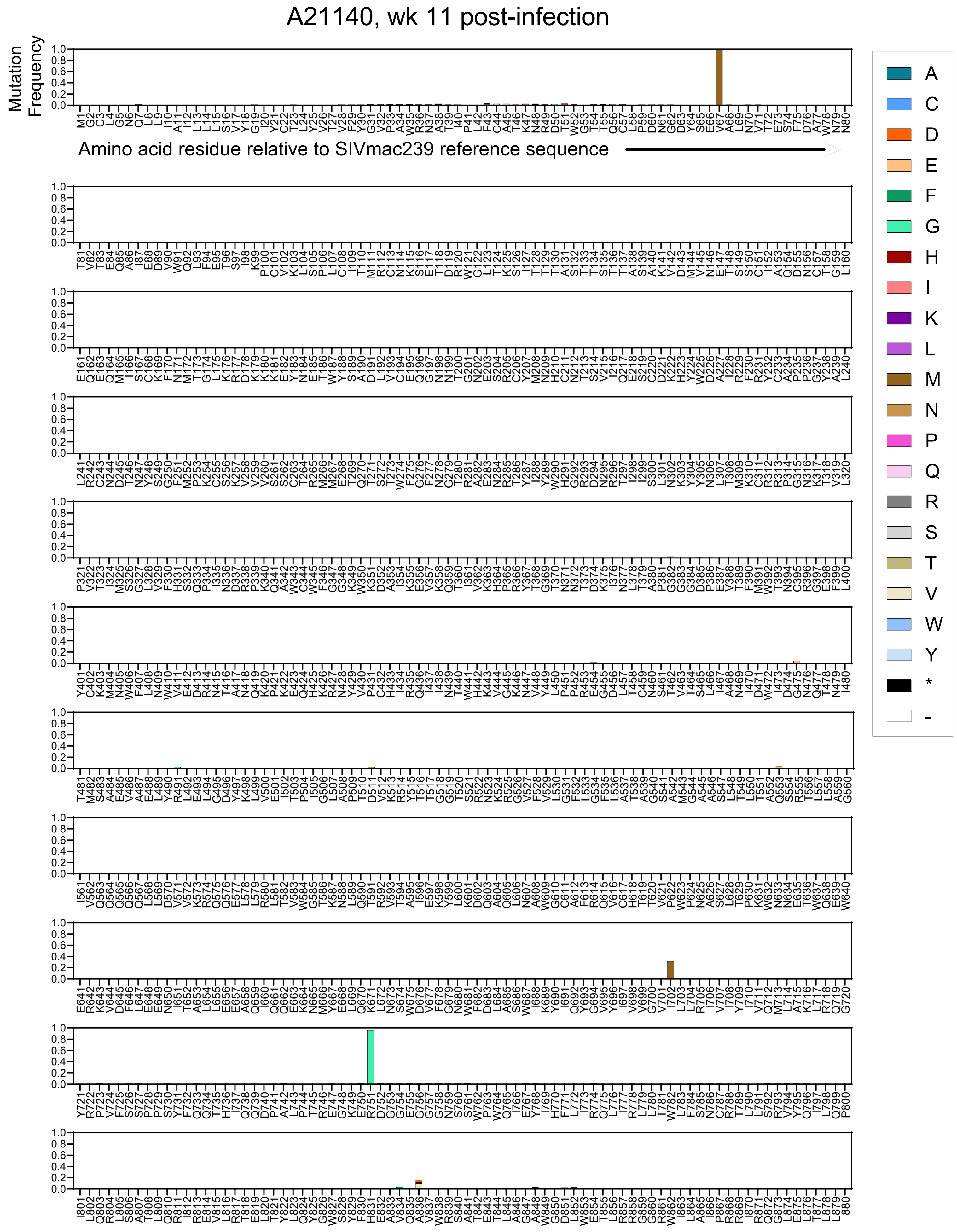

Fig.S21. A21140 Plasma SIV Env sequences. Plasma SIVmac239 viruses from 11 weeks post-infection were sequenced for the Env gene by long-read sequencing. Open reading frames were aligned to the SIVmac239 reference sequence (GenBank: M33262) by an in-house deep sequencing analysis pipeline. Amino acid mutation frequencies are plotted, with each mutation indicated by a color corresponding to the legend insert.

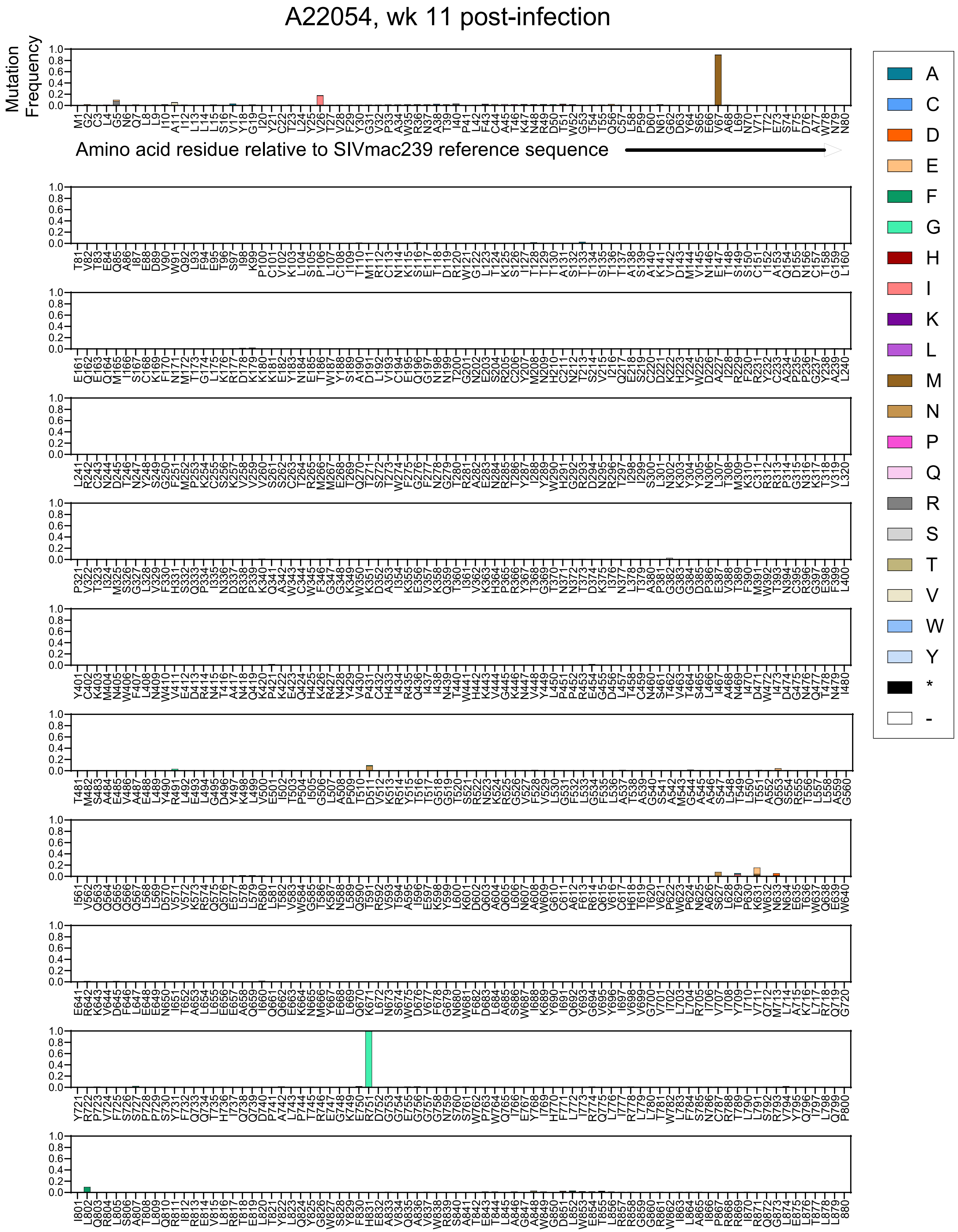

Fig.S22. A22054 Plasma SIV Env sequences. Plasma SIVmac239 viruses from 11 weeks post-infection were sequenced for the Env gene by long-read sequencing. Open reading frames were aligned to the SIVmac239 reference sequence (GenBank: M33262) by an in-house deep sequencing analysis pipeline. Amino acid mutation frequencies are plotted, with each mutation indicated by a color corresponding to the legend insert.
